# Lifespan regulation in α/β posterior neurons of the fly mushroom bodies by Rab27

**DOI:** 10.1111/acel.13179

**Published:** 2020-07-06

**Authors:** Wen‐Yu Lien, Yu‐Ting Chen, Yi‐Jhan Li, Jie‐Kai Wu, Kuan‐Lin Huang, Jian‐Rong Lin, Shih‐Ching Lin, Chia‐Chun Hou, Horng‐Dar Wang, Chia‐Lin Wu, Shu‐Yi Huang, Chih‐Chiang Chan

**Affiliations:** ^1^ Graduate Institute of Physiology College of Medicine National Taiwan University Taipei Taiwan; ^2^ Department of Biochemistry and Graduate Institute of Biomedical Sciences College of Medicine Chang Gung University Taoyuan Taiwan; ^3^ Institute of Biotechnology National Tsing Hua University Hsinchu Taiwan; ^4^ Department of Neurology Linkou Chang Gung Memorial Hospital Taoyuan Taiwan; ^5^ Department of Medical Research National Taiwan University Hospital Taipei Taiwan

**Keywords:** Drosophila, lifespan extension, mushroom body, Rab27, S6K, TOR

## Abstract

Brain function has been implicated to control the aging process and modulate lifespan. However, continuous efforts remain for the identification of the minimal sufficient brain region and the underlying mechanism for neuronal regulation of longevity. Here, we show that the *Drosophila* lifespan is modulated by *rab27* functioning in a small subset of neurons of the mushroom bodies (MB), a brain structure that shares analogous functions with mammalian hippocampus and hypothalamus. Depleting *rab27* in the α/βp neurons of the MB is sufficient to extend lifespan, enhance systemic stress responses, and alter energy homeostasis, all without trade‐offs in major life functions. Within the α/βp neurons, *rab27KO* causes the mislocalization of phosphorylated S6K thus attenuates TOR signaling, resulting in decreased protein synthesis and reduced neuronal activity. Consistently, expression of dominant‐negative S6K in the α/βp neurons increases lifespan. Furthermore, the expression of phospho‐mimetic S6 in α/βp neurons of *rab27KO* rescued local protein synthesis and reversed lifespan extension. These findings demonstrate that inhibiting TOR‐mediated protein synthesis in α/βp neurons is sufficient to promote longevity.

## Graphical abstract

The Drosophila lifespan is modulated by *rab27* functioning in α/βp neurons of the mushroom bodies, a brain structure functionally analogous to mammalian hippocampus and hypothalamus. Depleting *rab27* in these neurons is sufficient to extend lifespan and enhance systemic stress resistance without trade‐offs in major life functions.

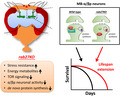

## 1 INTRODUCTION

The desire for lifespan extension is a never‐ending quest throughout human history. While extreme lifespans are likely limited genetically, environmental factors and acquired characteristics do affect the speed of degeneration and health span. As a result, lifespan is determined as the collective effect of internal and external factors, including the challenges of oxidative stress, the homeostasis of circulating metabolites, intake of energy and nutrients, and the allocation of resources among major life functions. Restriction of caloric intake is by far the most effective approach to extend lifespan in all species examined from yeast to non‐human primates. Although its long‐term effect is hard to evaluate in humans, caloric restriction has been shown to induce physiological changes that are similar to those observed in animal models. However, when energy intake is restricted, limited resources need to be relocated from growth and reproduction to maintain life‐sustaining functions, resulting in trade‐offs between longevity and the size/weight of an individual as well as the reproductive success (Maklakov & Immler, 2016).

Animals need to sense, integrate, and adapt to changes from diverse physiological and environmental cues. In the mammalian hypothalamus, different clusters of neurons integrate internal and external inputs to regulate important life functions including appetite, body temperature, and sleep. For example, the murine arcuate nucleus integrates hormonal signals from the periphery including ghrelin and leptin to regulate food intake for energy homeostasis. Two other hypothalamic nuclei, the preoptic area, and the dorsomedial hypothalamus integrate peripheral inputs to maintain body temperature. This evidence illustrates the importance of the central nervous system (CNS) for the summation of peripheral signals to regulate systemic homeostasis. Whether the CNS also functions as a control hub for lifespan regulation; however, remains debatable.

Several recent studies suggest that the hypothalamus plays a pivotal role in longevity and it may be a regulator of systemic aging (Zhang et al., 2013; Zhao et al., 2017). In the fruit fly *Drosophila*, the functions of mammalian hypothalamus are divided among the mushroom bodies (MB) and the pars intercerebralis (Bang et al., 2011; Dus et al., 2015). Within the pars intercerebralis, the median neurosecretory cluster (mNSC) has been shown to regulate lifespan, as the ablation of mNSC results in altered systemic energy metabolism, enhanced stress tolerance, and lifespan extension (Broughton et al., 2005). The MB consists of ~ 2000 Kenyon cells that can be classified according to the axon innervation patterns into three major groups: the α/β, α’/β’, and γ lobes. Although the MB is important for learning and memory similar to the hippocampus, the MB also shows functional analogy to the mammalian hypothalamus in regulating food‐seeking behavior, courtship behavior, sleep, and temperature preference (Bang et al., 2011; Joiner, Crocker, White, & Sehgal, 2006; McBride et al., 2005). For example, the *Drosophila* MB integrates hunger and satiety signals to regulate feeding behavior to meet organismal needs (Tsao, Chen, Lin, Yang, & Lin, 2018). Also, reduction of insulin signaling in the MB decreases food consumption (Zhao & Campos, 2012). However, whether the MB contributes to the regulation of lifespan remains unknown.

Rab27 is a highly conserved small Rab GTPase widely expressed in various secretory cells, including endocrine, cancer, and immune cells, and is well recognized for its role in exosome secretion. We have reported that the *Drosophila* Rab27 is exclusively expressed in neuronal tissues predominantly in specific brain regions including the MB (Chan et al., 2011; Jin et al., 2012). At present, the neuronal functions of Rab27 remain poorly understood. Rab27 regulates the docking of dense‐core vesicles in PC12 neuroendocrine cells (Tsuboi & Fukuda, 2006), whereas inhibition of Rab27 is found to impair synaptic transmission in *C. elegans* and giant squid (Mahoney et al., 2006; Yu et al., 2008). We have previously characterized *rab27KO* flies as viable and fertile, without apparent developmental defects (Chan et al., 2011). Here, we describe our investigation of how Rab27 functions to control lifespan in a small subset of neurons in the *Drosophila* MB and the underlying molecular mechanism.

## 2 RESULTS

### 2.1 Loss of *rab27* in adult neurons is sufficient to prolong lifespan

**Figure 1:**
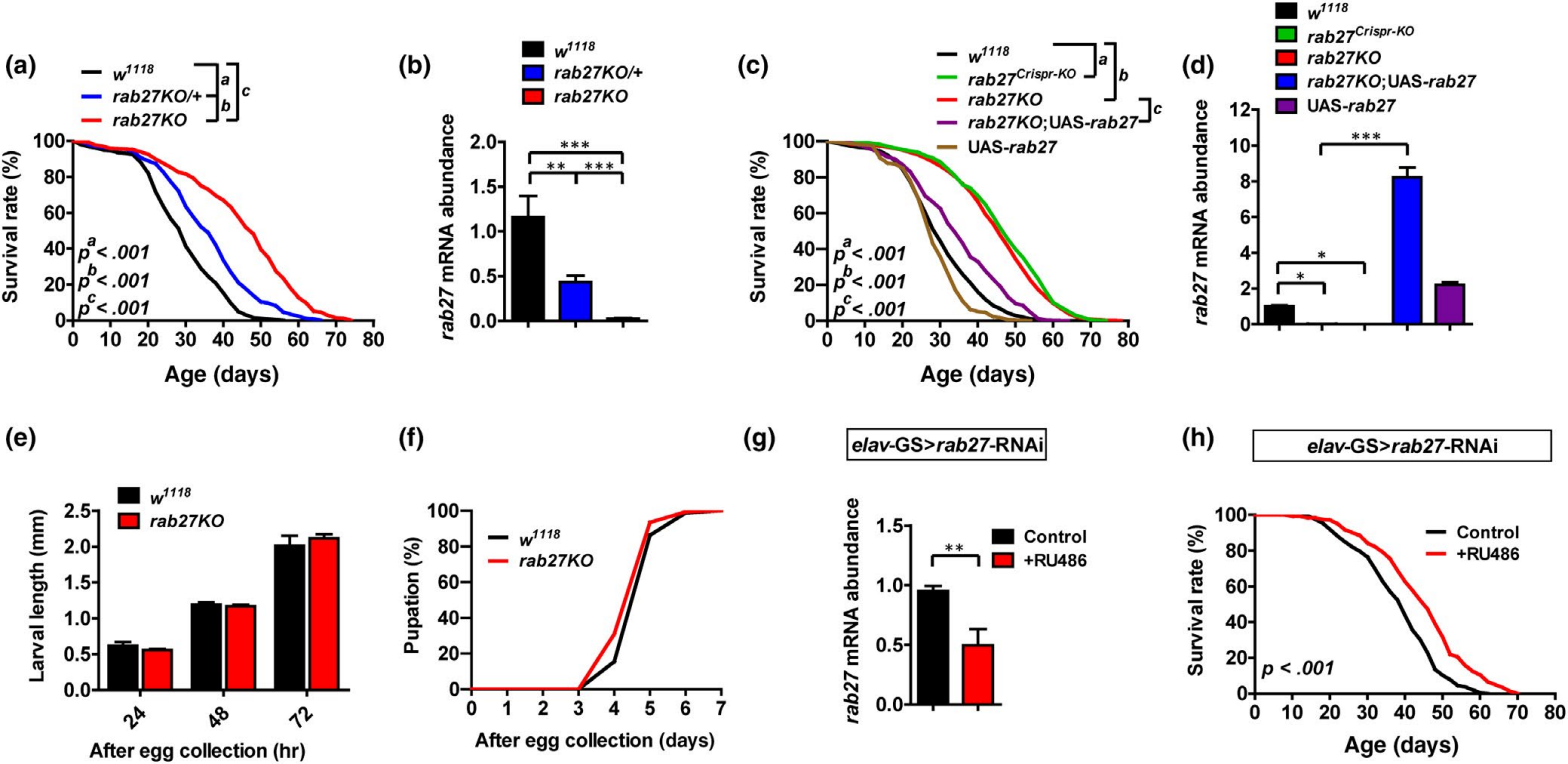
Loss of *rab27* in adult neurons is sufficient to prolong lifespan. (a) Survival of females from *w^1118^* control (black), *rab27* heterozygote *rab27KO/+* (blue), and homozygous *rab27KO* (red), *p < *.001. (b) Relative abundance of *rab27* mRNA in strains in (a) determined by qRT‐PCR in fly heads; mean ± *SEM* of three independent experiments. **p < .01, ***p < .001, one‐way ANOVA. (c) Survival of females from two independent *rab27* nulls, *rab27^Crispr‐KO^* (green), and *rab27KO* (red), comparing with *w^1118^* control (black) and *rab27KO*;UAS‐*rab27* (purple), *p < *.001. (d) Relative abundance of *rab27* mRNA in strains in (c) in fly heads. *p < .05, ***p < .001, one‐way ANOVA. (e) Body length measured at 24, 48, and 72 hr after egg collection, *n* = 3 independent experiments. (f) Developmental time from egg to pupa in *w^1118^* and *rab27KO*, *n* = 3 independent experiments. (g) Relative abundance of *rab27* mRNA in the heads of *elav*‐GS‐Gal4 > UAS‐*rab27*‐RNAi flies fed with RU486 normalized to the solvent‐fed control (EtOH). *p* < .01, significance determined by one‐way ANOVA, also for Gal4 or UAS, controls is shown in Figure S2e. (h) Survival of females from RU486‐induced *elav*‐GS‐Gal4 > UAS‐*rab27*‐RNAi (red) compared with the solvent‐fed control (black), *p* < .001. All survival data were analyzed by log‐rank tests. Please see Tables S1 and S2 for detailed information including mean lifespan and statistical comparisons

We have shown that *rab27^Gal4‐KO^* homozygotes are viable (this strain was used for most experiments unless otherwise noted and is herein referred to as *rab27KO*) (Chan et al., 2011). Characterizing these flies in detail, we found that the homozygous *rab27KO* flies showed a pronounced lifespan extension compared with wild‐type (WT) controls (average survival + 46.6%) and *rab27* heterozygotes also had an intermediate yet significant lifespan extension in the population‐based longevity assay (Figure 1a,b, average survival + 20.3%; (Flatt et al., 2008); all of the lifespan statistics of the female were shown in the following figures and summarized in Table S1, whereas those of the male were summarized in Table S2). We generated another independent null allele of *rab27* using CRISPR/cas9 (*rab27^Crispr‐KO^*, Figure S1a,b) which showed the same effect on lifespan (Figure 1c,d, average survival + 53.5%). These two *rab27* knockout strains were further validated by the single‐sex vial assays (Bjedov et al., 2010), and they exhibited similar trends of lifespan extension (Figure S1c). The effect of *rab27KO* on lifespan was reversed by the expression of *rab27* cDNA, confirming that *rab27* regulates longevity (Figure 1c,d). Thus, the global loss of *rab27* prolongs lifespan in *Drosophila*.

We have shown that Rab27 is expressed throughout life (Chan et al., 2011; Jin et al., 2012). We observed no difference in larval body length and pupation timing between WT and *rab27KO* (Figure 1e,f), suggesting that the lifespan extension is less likely due to developmental defects. To determine whether depleting *rab27* after the flies reach the adult stages still promotes longevity, we utilized an RU486‐inducible system for pan‐neuronal *rab27* RNAi after eclosion. Feeding of RU486 to adult flies leads to effective pan‐neuronal activation of *elav*‐GS‐Gal4 (Figure S2a‐d’’), lowered *rab27* mRNA abundance in the head (Figure 1g and Figure S2e), and significantly prolonged lifespan compared with the non‐fed control (Figure 1h, average survival + 17.1% and Figure S2f). Thus, reducing *rab27* mRNA in the adult brain is sufficient to extend lifespan.

### 2.2 *rab27* controls systematic stress response and energy metabolism

**Figure 2:**
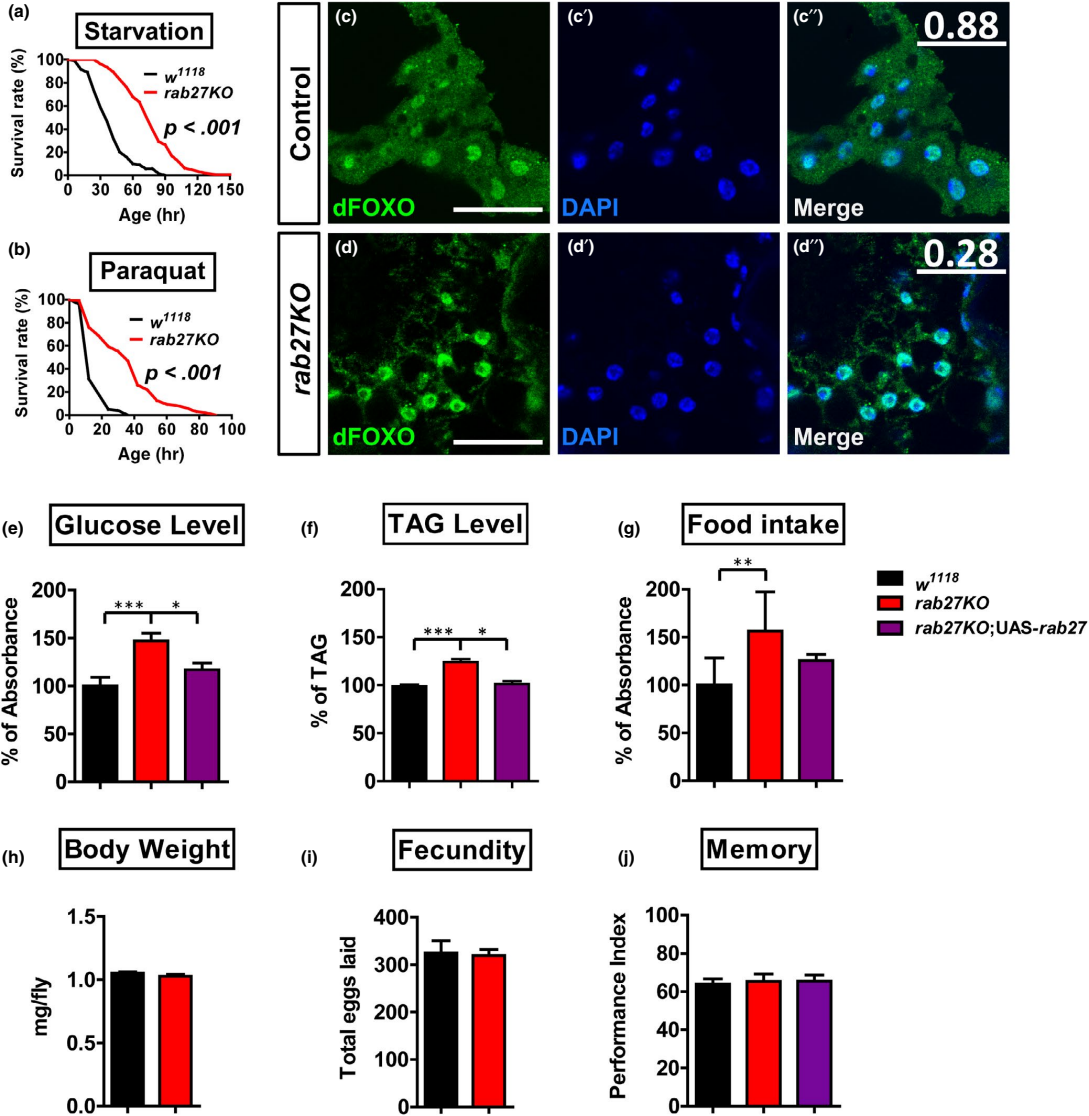
*rab27KO* flies lived longer under stress and had altered metabolic homeostasis. (a–b) Survival of females from *w^1118^* and *rab27KO* under (a) starvation (*p* < .001) or (b) paraquat‐induced reactive oxygen species stress (*p* < .001). (c–d’’) Representative confocal fluorescence images of the subcellular distribution of endogenous dFOXO (green) in the abdominal fat body of WT (c–c’’) or *rab27KO* (d–d’’). Underlined numbers at the upper right corner (c”, d”) indicate the ratio of cytoplasmic‐to‐nuclear dFOXO signal (*n* = 3; *p* < .05). Nuclei are labeled by DAPI (blue). Scale bars: 25 µm. (e) Levels of circulating glucose in females normalized to *w^1118^*, *p < .05, ***p < .001, *n* = 3 independent experiments. (f) Levels of TAG in whole female animals, normalized to both the body weight and the level in *w^1118^*. *p < .05, ***p < .001, *n* = 3 independent experiments. (g) Levels of food intake normalized to *w^1118^*. *p* < .01, *n* = 10 independent experiments. (h) Body weight of 21‐day‐old female *w^1118^* and *rab27KO* flies, *n* = 70 flies per condition. (i) Quantification of female fecundity shown by accumulated eggs laid from day 7 to day 42 post‐eclosion. *n* = 20 flies per condition. (j) The performance index of an olfactory associative learning assay testing 7‐day‐old flies of the indicated genotypes. *n* = 3 independent experiments

Many long‐lived mutants are reported to be highly resistant to oxidative stress and starvation. Indeed, we found that *rab27KO* flies lived longer than WT even under starvation or paraquat‐induced oxidative stress (Figure 2a,b). Also, we observed a similar increase in the lifespan of *rab27KO* under starvation in an isogenized Canton‐S background (Figure S3a), suggesting that the extension effect is independent of genetic backgrounds. Moreover, the levels of *Wolbachia* DNA in our experimental strains were negligible (Figure S3b) and the longevity of *rab27KO* flies remained after 3 generations of tetracycline treatment (Figure S3c), demonstrating that the effect of *rab27KO* on longevity was not related to *Wolbachia* infection, which has been shown to reduce the lifespan in *Drosophila* (Min & Benzer, 1997).

The nuclear translocation of Forkhead box class O (dFOXO) in the peripheral tissues of *Drosophila* is associated with an extended lifespan, increased stress resistance, and altered lipid metabolism (Hwangbo, Gershman, Tu, Palmer, & Tatar, 2004). The activation of dFOXO in the fat body, as indicated by its nuclear localization, is a common feature linked to extended lifespan (Martins, Lithgow, & Link, 2016). We thus examined the subcellular dFOXO localization in adult fat bodies, a tissue that does not express *rab27*. In the *rab27KO* fat bodies, we found a reduced ratio of cytoplasmic‐to‐nuclear dFOXO compared with that of WT control (Figure 2c‐d’’), suggesting a cell non‐autonomous effect of *rab27KO*. In *rab27KO* adults, the levels of circulating glucose, TAG, and food intake were significantly higher than those in WT controls, and the increases in metabolites could be reversed by *rab27* expression (Figure 2e‐g). Notably, while many long‐lived mutants are known to prolong lifespan at the expense of body size/weight and fecundity (Bai, Post, Kang, & Tatar, 2015; Bjedov et al., 2010; Gronke, Clarke, Broughton, Andrews, & Partridge, 2010), *rab27KO* flies exhibited comparable weight and fecundity with WT (Figure 2h,i). Also, the olfactory memory of *rab27KO* flies was equivalent to that of WT (Figure 2j). We concluded that removing *rab27* increases resistance to starvation and ROS stress and affects specific aspects of energy metabolism.

### 2.3 Reduction of *rab27* in the α/β posterior (α/βp) neurons of the MB increases longevity

**Figure 3:**
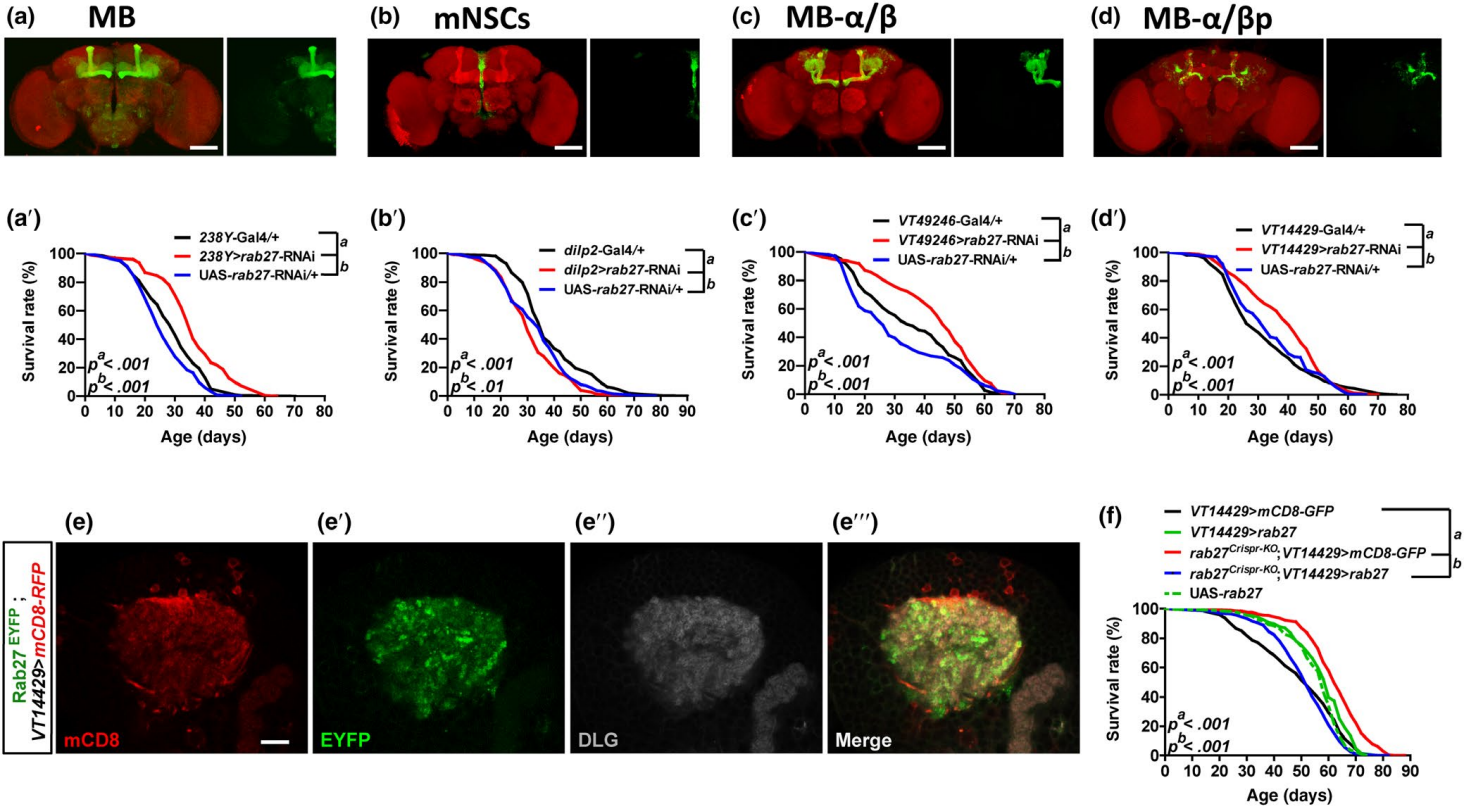
Reducing *rab27* in the α/β posterior (α/βp) neurons of the mushroom bodies (MB) increases longevity. (a–d) Expression patterns of Gal4 lines in the brain visualized by UAS‐*mCD8‐GFP* (green). The brain was stained with anti‐Disks large (DLG) to label general neuropils (red). Scale bars: 75 µm. (a’–d’) Survival of females from *rab27* knockdown strains (red) compared with the corresponding Gal4 (black) and UAS (blue) lines, including *rab27* knockdown in (a’) the MB (*238Y* > *rab27*‐RNAi), *p* < .001; (b’) the mNSCs (*dilp2* > *rab27*‐RNAi),* p* < .01; (c’) α/β lobes of the MB (*VT49246* > *rab27*‐RNAi), *p* < .001; and (d’) α/βp neurons of the MB (*VT14429* > *rab27*‐RNAi),* p* < .001. (e–e’’’) Comparison of Expression of Rab27^EYFP^ (green) from the endogenous locus versus. *VT14429*‐Gal4‐driving UAS‐*mCD8‐RFP* (red). Anti‐DLG labels the post‐synaptic dendritic termini (gray). Scale bars: 10 µm. (f) Survival of males from *VT14429* > *mCD8‐GFP* (black), *VT14429* > *rab27* (green, solid), *rab27^Crispr‐KO^*;*VT14429* > *mCD8‐GFP* (red), *rab27^Crispr‐KO^*;*VT14429* > *rab27* (blue), UAS‐*rab27* (green, dashed). *p < *.001. All survival data were analyzed by log‐rank tests

Rab27 protein is only detected in neurons (Jin et al., 2012). We verified this finding by examining *rab27* expression in the whole body and identified specific enrichment in the MB, mNSC, and subesophageal ganglion (SOG) neurons of the brain (Figure S4a‐d and Figure S5a). To determine which subset of neurons requires *rab27* to mediate lifespan regulation, we performed *rab27* RNAi with a series of Gal4 lines that drives expression in distinct brain regions (the Gal4 expression patterns are shown in Figure 3a‐d and Figure S5a‐d; summarized in Figure S4e). Reduction of *rab27* mRNA in all *rab27*‐expressing cells (Figure S5a’, average survival + 12.7%) or the MB neurons (Figure 3a’, average survival + 23.3%), but not the mNSC or the SOG (Figure 3b’and Figure S5b’), extended lifespan compared with the controls. Within the MB, knocking down *rab27* in the α/β lobes leads to a modest but significant increase of lifespan (Figure 3c’, average survival + 17.5%), whereas reducing *rab27* in the α’/β’ lobes or γ lobe did not (Figure S5c’,d’). Interestingly, lowering *rab27* mRNA in the α/βp region, which contains only ~ 73 neurons (Figure S6a,b; (Aso et al., 2009)), was sufficient to extend lifespan (Figure 3d’, average survival + 13%). We examined the expression of an endogenously tagged Rab27^EYFP^ (Dunst et al., 2015) and confirmed that Rab27 was indeed detected in the axons and dendrites but not the cell bodies of the α/βp neurons (Figure 3e‐e’” and Figure S6c‐d’’). Importantly, expressing *rab27* cDNA in the α/βp neurons significantly reversed the lifespan extension of *rab27^Crispr‐KO^* (Figure 3f). In sum, our data indicate that the α/βp neurons of MB regulate lifespan via a *rab27*‐dependent mechanism.

### 2.4 The α/βp neurons are required for lifespan maintenance

We next examined the requirement and mechanism of the α/βp neurons in lifespan regulation. Expression of the pro‐apoptotic gene *reaper* in the α/βp neurons in the WT background shortened the lifespan (Figure S7a), indicating that the α/βp neurons are essential to lifespan control. Genetic ablation of these neurons with the cytotoxic protein Ricin by 3 independent Gal4 lines led to larval lethality (Figure S7b), possibly due to a general toxic effect during development. In contrast, ablation of γ lobe wherein *rab27* expresses does not affect viability. To better elucidate whether the α/βp neurons are required to regulate lifespan, we bypassed larval development by applying Gal80^ts^ to inhibit *ricin* expression at 18℃ until eclosion. We shifted adult flies to 29℃ to allow Ricin expression in the α/βp neurons, and the lifespan was significantly shortened (Figure S7c). Thus, the α/βp neurons are required for the maintenance of lifespan.

**Figure 4:**
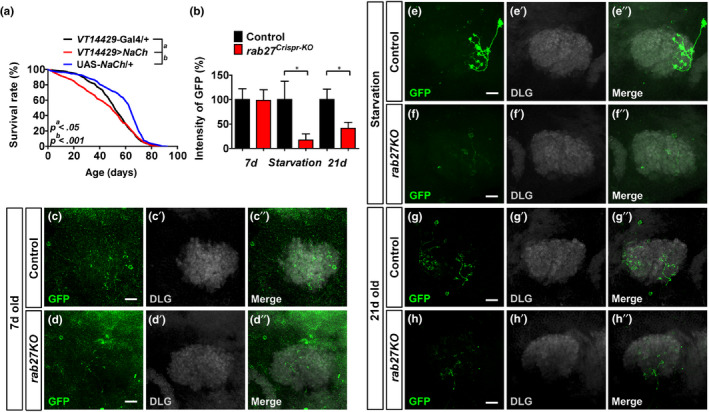
Reduced α/βp neuronal activity in *rab27KO* flies corresponds with lifespan extension. (a) Survival of female flies overexpressing the sodium channels (*VT14429* > *NaCh*) compared with controls (UAS*‐NaCh*/+ and *VT14429*‐Gal4/+), *p < .05, ***p < .001, log‐rank tests. (b) Quantification of GFP fluorescence intensity in the α/βp neurons of male flies. Data are represented as mean ± *SEM* measured in at least five brains, *p* < .05. (c–h’’) Representative confocal fluorescence images of the α/βp neurons of flies bearing the *VT14429*‐Gal4 > *LexAop‐CD2‐GFP* and UAS‐*mLexA‐VP16‐NFAT*, *LexAop‐CD2‐GFP* transgenes. (c–d”) control group; (e–f”) under 1.5 days of starvation; and (g–h’’) at day 21 post‐eclosion. The area labeled with anti‐DLG showed the post‐synaptic, dendritic termini (gray). Scale bar: 10 µm

Rab27 participates in exocytosis in worms and mammalian cells (Mahoney et al., 2006; Tsuboi & Fukuda, 2006); therefore, we asked whether neurosecretion mediates the effect of the α/βp neurons on lifespan. We forced membrane depolarization thus enhanced neurosecretion by activating the α/βp neurons with overexpression of sodium channel (Geminard, Rulifson, & Leopold, 2009) and found a slight but significant decrease in lifespan (Figure 4a). To reveal whether the neuronal activity of the α/βp neurons is affected by *rab27*, we utilized the CaLexA (calcium‐dependent nuclear entry of LexA) system, wherein GFP intensity correlates with depolarization‐induced calcium influx (Masuyama, Zhang, Rao, & Wang, 2012). Comparing to WT, the GFP intensity was lower in the α/βp neurons of *rab27KO* flies after either 1.5 days of starvation (Figure 4b‐f”) or 3 weeks of aging (Figure 4b‐d’’ and Figure 4g‐h”), indicating a reduction in neuronal activity when *rab27KO* animals were stressed or aged.

### 2.5 *rab27* deactivates TOR signaling in the α/βp neurons to extend longevity

**Figure 5:**
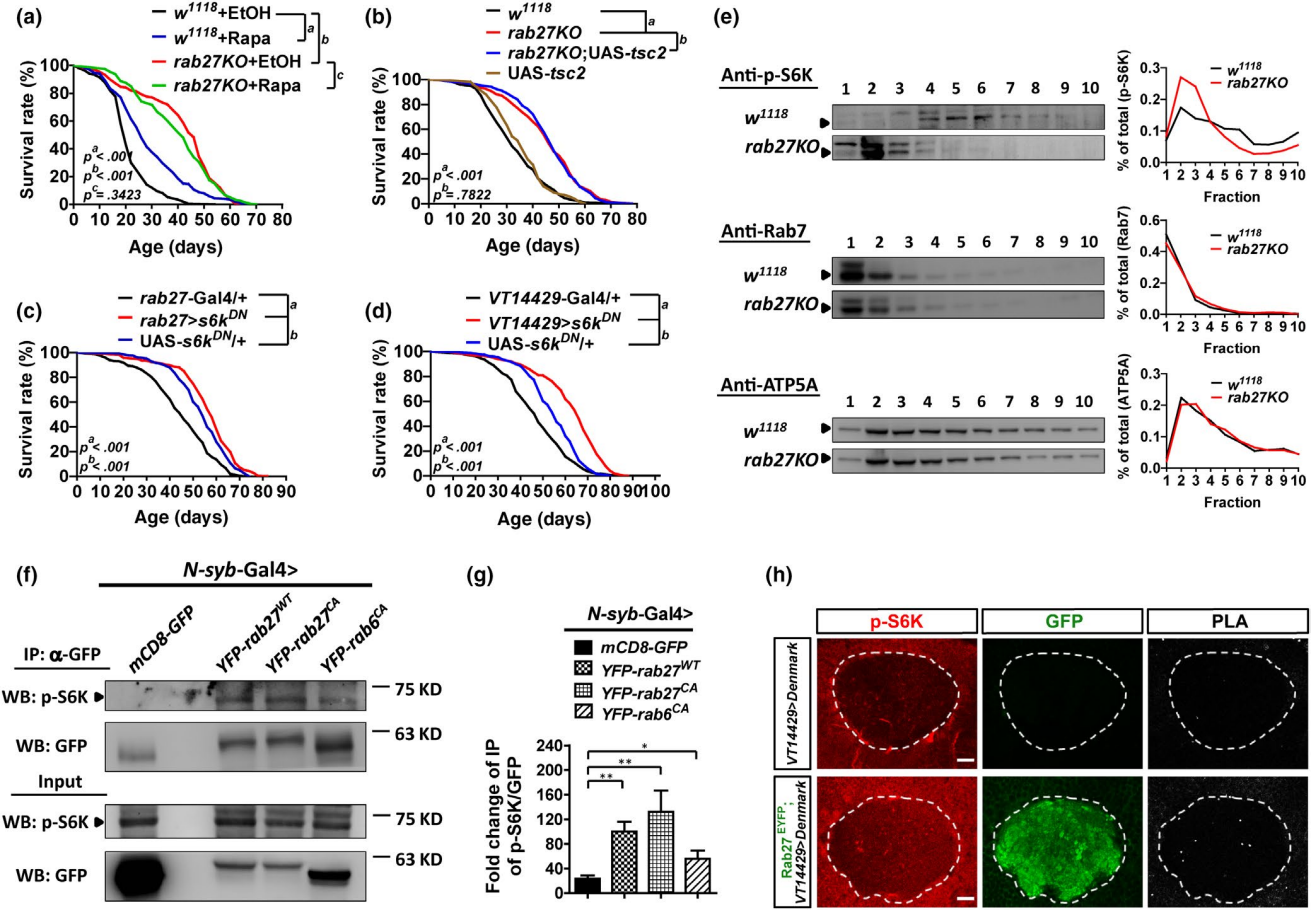
*rab27* interacts with TOR signaling to modulate lifespan. (a) Survival of females from *rab27KO* fed with Rapamycin (Rapa) compared with controls EtOH‐fed *rab27KO*, Rapa‐fed *w^1118^*, and EtOH‐fed *w^1118^*, *p < *.001. Notably, EtOH feeding causes significant shortening in lifespan. (b) Survival of females from *rab27KO* expressing *tsc2* compared with controls (*w^1118^* and *rab27KO*), *p < *.001. (c) Survival of females from *rab27‐*Gal4 > UAS‐*s6k^DN^* with controls (UAS*‐s6k^DN^*/+ and *rab27‐*Gal4/+), *p < *.001. (d) Survival of females from *VT14429‐*Gal4 > UAS‐*s6k^DN^* compared with controls (UAS*‐s6k^DN^*/+ and *VT14429‐*Gal4/+), *p < *.001. All survival data were analyzed by log‐rank tests. (e) Subcellular fractionation of *w^1118^* and *rab27KO* head lysates immunoblotted with p‐S6K, Rab7 (late endosomal marker), and ATP5A (mitochondrial marker). The quantification blots represent the means of 5 individual experiments. (f) Immunoprecipitation of head lysates with the expression of YFP‐tagged Rab27^WT^, constitutively activated Rab27 (Rab27^CA^), or constitutively activated Rab6 (Rab6^CA^) with an anti‐GFP antibody. The resulting samples were analyzed by immunoblotting with anti‐p‐S6K antibody. The number indicates the ratio of p‐S6K to GFP/YFP in the IP fraction. (g) Quantification of co‐IP of p‐S6K with YFP‐Rabs. *p < .05, **p < .01, one‐way ANOVA. (h) The in vivo association of Rab27^EYFP^ and p‐S6K determined by the Proximity Ligation Assay (PLA). The brains were stained with anti‐GFP antibodies to detect Rab27^EYFP^ (green) and anti‐p‐S6K antibodies (red), followed by the addition of PLA‐specific probes to detect proximity ligation events (gray). White circles mark the post‐synaptic, dendritic area of the α/βp neurons. Scale bars: 7.5 µm

A wealth of literature has shown that TOR signaling mediates neuronal activity. *rab27* knockout phenocopies flies of reduced TOR activity in several aspects, including lifespan extension (Figure 1 versus. (Kapahi et al., 2004)), stress resistance (Figure 2a, b versus. (Bjedov et al., 2010)), and TAG accumulation (Figure 2f versus. (Bjedov et al., 2010)). We then examined the genetic interplays between *rab27* and components of the TOR pathway. The lifespan of *rab27KO* flies was not further extended when we inhibited TOR signaling by Rapamycin feeding (Figure 5a) or *tsc2* overexpression (Figure 5b). Thus, *rab27* likely functions through the TOR pathway. The phosphorylation of ribosomal protein S6 (p‐S6) by S6 kinase is an important downstream readout of TOR activity. We then deactivated TOR signaling by expressing a dominant‐negative form of the S6 kinase (*s6k^DN^*) specifically in *rab27*‐expressing cells or in the α/βp neurons. Interestingly, suppressing TOR activity in these neurons was sufficient to increase longevity (Figure 5c,d, average survival + 5.2% and + 14.2%, respectively), supporting that the effect of *rab27KO* on lifespan is mainly mediated via the TOR pathway. Besides S6K, the *Drosophila* 4E‐BP (*thor*) is another target downstream of the TOR signaling pathway. Overexpression of *thor* in muscle has been shown to result in lifespan extension in flies (Demontis & Perrimon, 2010). Here, we showed that *thor* overexpression in the α/βp neurons did not extend lifespan (Figure S8). Altogether, *rab27* deactivates TOR signaling for lifespan extension likely through an *s6k*‐specific but *thor*‐independent pathway.

### 2.6 Rab27 binds with p‐S6K and determines its subcellular localization

Because many Rab proteins are known regulators of vesicle trafficking, we performed sucrose gradient centrifugation of fly head extracts to determine whether Rab27 regulates the localization of TOR pathway components. In the head extracts of Rab27^EYFP^ flies, Rab27 co‐fractionated with phosphorylated S6K (p‐S6K) proteins (Figure S9). Compared with the WT control, the distribution of p‐S6K changed in *rab27KO* (Figure 5e), whereas two control proteins, Rab7 and ATP5A, showed no differences, suggesting that *rab27KO* did not abolish global protein distribution but specifically affected the subcellular localization of p‐S6K. Also, we found that p‐S6K co‐immunoprecipitated with Rab27 (Figure 5f,g) and confirmed the interaction in vivo by a Proximity Ligation Assay (PLA) (Mosca, Luginbuhl, Wang, & Luo, 2017) (Figure 5h), demonstrating a direct association between Rab27 and p‐S6K in the α/βp neurons. Altogether, these results indicate that Rab27 may regulate the subcellular localization of p‐S6K in *Drosophila* brains.

### 2.7 Rab27 anchors activated S6K to the periphery of α/βp neurons for de novo protein synthesis

**Figure 6:**
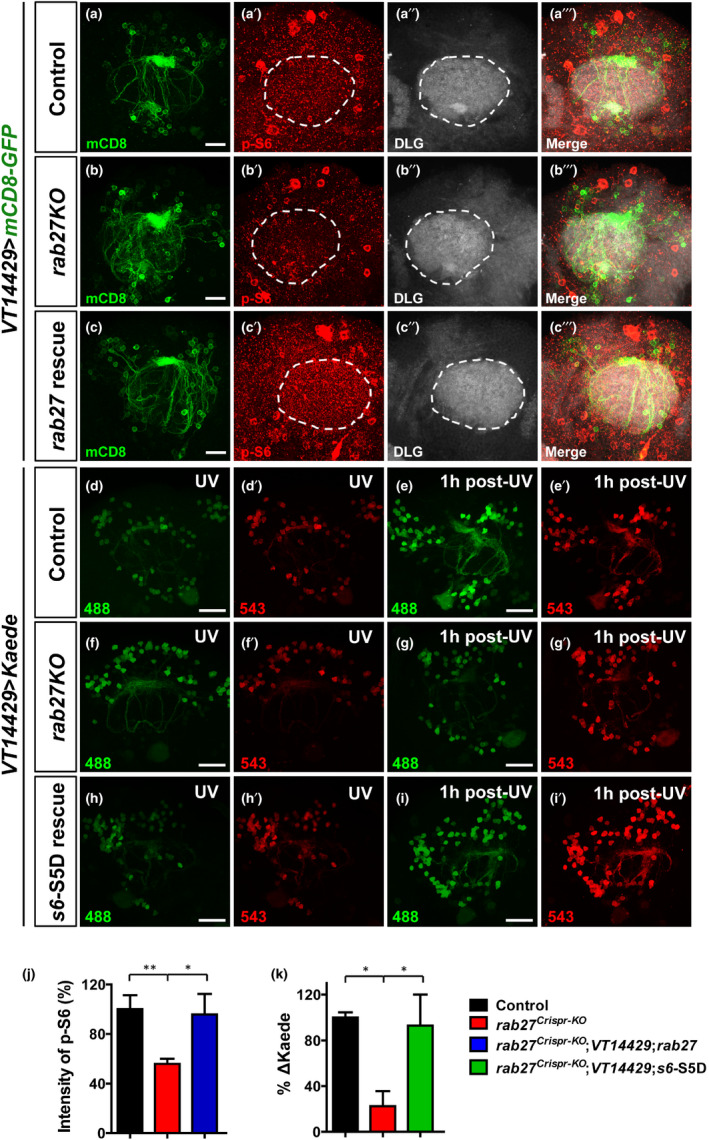
Rab27 anchors p‐S6K to the periphery of the α/βp neurons for de novo protein synthesis. (a–c’’’) Representative confocal fluorescence images of the dendrites of the α/βp neurons expressing UAS‐*mCD8‐GFP* (green) stained with anti‐p‐S6 (red) and DLG (post‐synaptic marker, gray) in *VT14429‐*Gal4 > UAS‐*mCD8‐GFP* (a‐a’’’), *rab27 ^Crispr‐KO^*;*VT14429*‐Gal4 > UAS‐*mCD8‐GFP* (b–b’’’) or *rab27^Crispr‐KO^*;*VT14429*‐Gal4 > UAS‐*rab27*, UAS‐*mCD8‐GFP* (c–c’’’). Scale bars: 10 µm. (d–i’) Tracing de novo protein synthesis in the α/βp neurons with photoconvertible Kaede protein. Adult *VT14429‐*Gal4 > UAS‐*Kaede* or *rab27^Crispr‐KO^*;*VT14429*‐Gal4 > UAS‐*Kaede* animals were exposed to ultraviolet light (UV) for 6 hr (d,f) to irreversibly convert all existing Kaede to red fluorescence (d’,f’). Newly synthesized green Kaede protein was examined 1 hr after UV exposure in *VT14429‐*Gal4 > UAS‐*Kaede* (e‐e’) or *rab27^Crispr‐KO^*;*VT14429*‐Gal4 > UAS‐*Kaede* (g–g’). The strain for rescue experiments: *rab27^Crispr‐KO^*;*VT14429*‐Gal4 > UAS‐*s6*‐S5D, UAS‐*mCD8‐GFP* (h–i’). Scale bars: 25 µm. (j) Quantification of p‐S6 intensity by marking DLG positive areas and measured the red signal. The fluorescence intensity was normalized to the control levels. Data are represented as mean ± *SEM* measured in at least three brains, *p < .05, **p < .01. (k) Quantification of de novo protein synthesis expressed as the difference in green Kaede protein levels (ΔKaede) immediately after photoconversion and 1‐hr post‐UV. The ΔKaede level in *rab27^Crispr‐KO^* was normalized to the WT levels. Data are represented as mean ± *SEM* measured in at least three brains, **p < .01

We then investigated the subcellular localization of Rab27 to further understand its function in the α/βp neurons. Since Rab27 has long been recognized to function in cargo docking during exocytosis (Fukuda, 2006; Kasai et al., 2005), we speculated that Rab27 may serve as an anchor to mediate the transport of S6K. While Rab27 was only detected in the axons and dendrites but not the cell bodies of the α/βp neurons (Figure 3e‐e’” and Figure S6c‐d’’), the levels of p‐S6K in the dendrites and axons were both reduced in *rab27KO* (Figure S10). Also, the level of p‐S6 was decreased in the dendrites in *rab27^Crispr‐KO^* (Figure 6a‐b’” and Figure 6l). Also, we detected a significant increase in p‐S6 level by *rab27* expression specifically in the α/βp neurons of *rab27KO* (Figure 6c‐c’” and Figure 6l). The effect of *rab27* on de novo protein synthesis in α/βp dendrites was monitored by Kaede, a photoconvertible fluorescent protein that irreversibly turns from green to red upon UV irradiation. Loss of *rab27* does not affect Kaede maturation, as shown by the baseline green fluorescence compared with WT control (Figure S11). We then UV‐converted the majority of existing Kaede to red and then examined the presence of newly synthesized Kaede green protein in the α/βp neurons 1 hr post‐UV irradiation. In the α/βp neurons of *rab27KO* animals, the amount of de novo Kaede synthesis was significantly lower compared with that in WT controls (Figure 6d‐g’ and Figure 6m), and the reduction was rescued by *s6*‐S5D expression (Figure 6h‐k’ and Figure 6m). The extended longevity of *rab27KO* was not affected by the expression of a constitutively activated S6K (*s6k^CA^*) (Figure S12a). In contrast, the lifespan was significantly reversed by the expression of phospho‐mimetic *s6*‐S5D specifically in the α/βp neurons of *rab27KO* (Figure S12b). As a control, overexpression of *rab27* or phospho‐mimetic *s6*‐S5D in the WT α/βp neurons did not reduce lifespan (Figure S12c). Therefore, loss of *rab27* reduced protein synthesis in the α/βp neurons, likely as the consequence of mislocalized p‐S6K and reduced phosphorylation of S6.

## 3 DISCUSSION

Our data show that depleting *rab27* in the α/βp neurons of the *Drosophila* MB results in significant lifespan extension. Knockdown of *rab27* in these ~ 73 neurons was sufficient to cause systematic effects including altered homeostasis of key metabolites and the nuclear translocation of dFOXO in the fat body. Not only are *rab27KO* flies more resistant to starvation or oxidative stress, but they also show no detectable trade‐offs in weight, fecundity. Of note, adult *rab27KO* flies are not defective in olfactory memory at 1 week, although we could not rule out the possible acceleration in decline at older age. *rab27* modulates lifespan through the TOR pathway because 1) p‐S6K was mislocalized in *rab27KO* neurons resulting in reduced protein synthesis in α/βp neurons and diminished the neuronal activity; and 2) like *rab27KO*, expressing *s6k^DN^* in the α/βp neurons was also sufficient to extend lifespan. Together, our findings suggest that Rab27 functions upstream of S6 for protein synthesis in a specific small group of brain neurons to control longevity in *Drosophila*.

We observed the nuclear translocation of dFOXO in the fat body, wherein *rab27* is not expressed. The nuclear localization of dFOXO in the fat body is a common feature of lifespan extension, as it alters the downstream gene transcription, likely through the transcriptional regulation of longevity pathways such as stress resistance (Cathy Slack, Giannakou, Foley, Goss, & Partridge, 2011). Hwangbo *et al*. have shown that overexpression of dFOXO in adult fat bodies increased its nuclear localization and result in lifespan extension (Hwangbo et al., 2004). We show that *rab27KO* alleles exert non‐cell autonomous effects on dFOXO nuclear localization in the fat body, suggesting that *rab27* may act in a brain–fat body axis to elicit lifespan extension, thus highlighting the systemic effect of *rab27KO* on longevity. However, the molecular identity through which the *rab27*‐expressing neurons exert the cell non‐autonomous regulation on lifespan remains an open question.

The evolutionary fitness of an individual organism is best defined by the balance between survival and other major life functions such as reproduction. From an organismal perspective, a shift in energy expenditure from reproduction is often required to extend lifespan. Indeed, many studies have suggested trade‐offs between lifespan and reproduction, and many long‐lived mutants, such as single‐gene mutants of the TOR or insulin pathways, produce less offspring (Hansen, Flatt, & Aguilaniu, 2013; Partridge, Gems, & Withers, 2005). Growth is another life function that is highly associated with longevity from flies to monkeys (Mattison et al., 2017). For example, mutants of *chico*, which encodes the insulin receptor substrate in *Drosophila*, are long‐lived but have a metabolic imbalance and small body size (Bohni et al., 1999). However, the link between lifespan and body weight seems to be more complicated, as Slack et al. (2015) have shown that a *chico* mutation that disrupts its interaction with Grb2/Drk extends lifespan without affecting body weight (Slack et al., 2015). Moreover, a mutation in the odorant receptor *Or83b^2^* increases TAG level, enhances starvation tolerance, and extends lifespan with no effect on body weight or female fecundity (Libert et al., 2007). These reports and our findings all point to the possibility to uncouple lifespan control from the performance in essential life functions measured in this study. We noticed that the aforementioned phenotypes, such as glucose level, TAG level, food intake, and stress tolerance, are detected in aged flies but not young ones. There are two possibilities. For one, Rab27 may be only required at old age so that r*ab27KO* only causes physiological declines that are significant in old flies. Alternatively, the minute phenotypical differences that are below detectable levels at younger ages may accumulate over time and become obvious upon aging.

Most studies have attempted to modulate longevity at the level of individual animals. For example, systematic inhibition of the TOR signaling has been shown to extend lifespan from yeast to mammals. However, since the TOR pathway also regulates cell growth, ribosome biogenesis, and the process of translation, systemic inhibition of TOR signaling may come with a wide range of complications such as stomatitis, diabetes, and nephrotoxicity (Kaplan, Qazi, & Wellen, 2014). Instead of systematic manipulation, one plausible strategy to avoid potential complications is to identify the minimal region required to promote longevity. The brain serves as a good candidate since it is the integration center of input and output signals and can regulate a wide range of physiological functions. Previous reports have indicated that, in the *Drosophila* brain, the mNSC regulates systemic insulin levels by producing Dilps to mediate organism growth and lifespan (Broughton et al., 2005; Rulifson, Kim, & Nusse, 2002). Ablating mNSC leads to lifespan extension (Broughton et al., 2005), and limiting the secretion of Dilps from within the mNSC is also sufficient to increase longevity (Bai, Kang, & Tatar, 2012). In this study, we find that the α/βp neurons of the MB regulate systemic metabolism and lifespan. The *Drosophila* MB has been compared with the human hippocampus and hypothalamus based on functional analogy. Both the reduction of ROS levels in the hippocampus and expression of Sirt1 in the hypothalamus are linked to increased longevity in mice (Hu et al., 2007; Satoh et al., 2013). Also, Yang *et al*. showed that TOR activity is elevated in the hypothalamic pro‐opiomelanocortin (POMC) neurons in old mice and is linked to altered protein expression and changes in body weight (S. B. Yang et al., 2012), but the effect on lifespan remains unknown. However, how the neurons of the mammalian hippocampus or hypothalamus affect lifespan remains to be investigated. In this study, we show that *rab27KO* reduced the activity of a subset of MB neurons in aged flies, implying that manipulation of neuronal activity in a small number of neurons may have a profound effect on lifespan.

Here, we have shown that activating the α/βp neurons reduces the lifespan (Figure 4a), while the loss of *rab27* decreases neuronal activity (Figure 4b) and extends lifespan. We also provide evidence that *rab27KO* causes the mislocalization of S6K thus suppresses the phosphorylation of S6 in the dendrites of α/βp neurons of adult flies. Importantly, *s6*‐S5D expression in these neurons fully rescued the extended lifespan and reduced protein synthesis in *rab27KO*, suggesting that restoring protein synthesis suppresses α/βp‐mediated longevity. What is the mechanism linking S6K‐dependent protein synthesis to the neuron activity of α/βp and ultimately affecting lifespan? Reduction in localized protein translation has been shown to modulate neuronal function/activity. For example, localized protein synthesis at hippocampal synapses regulated by the neurotrophic factor BDNF is important for synaptic plasticity (Leal, Comprido, & Duarte, 2014). Regarding lifespan regulation, Zullo *et al*. recently found in *C. elegans* that inhibiting the excitability of glutamatergic or cholinergic neurons can increase longevity (Zullo et al., 2019). Also, Zhang *et al*. showed that the lifespan extension of *s6k* null mutant in *C. elegans* is suppressed by neuronal expression of *s6k* (Y. Zhang et al., 2019). While the nervous system of *C. elegans* is not organized into a brain with higher‐order structures and domains, our findings show that lifespan regulation by a small number of brain neurons may be an evolutionarily conserved phenomenon in the *Drosophila* brain, which shares functional analogous organization with higher organisms. In conjunction with these findings, we conclude that lifespan can be modulated by *rab27* in TOR‐mediated protein homeostasis in a small group of neurons. The consequence of reduced protein synthesis in the α/βp neurons of *rab27KO* remains to be investigated.

## 4 EXPERIMENTAL PROCEDURES

### 4.1 Key resources table

**Table:** 

Reagent or Resource	Source	Identifier
Antibodies		
Rabbit anti‐dFOXO	Cosmo Bio Co	Cat# CAC‐THU‐A‐DFOXO; RRID: AB_10705391
Mouse anti‐Drosophila discs large	Developmental Studies Hybridoma Bank (DSHB)	Cat# 4F3; RRID: AB_528203
Mouse anti‐Drosophila Bruchpilot (nc82)		Cat# nc82; RRID: AB_2314866
Mouse anti‐GFP		Cat# 12A6; RRID: AB_2617417
Alexa 568‐conjugated phalloidin antibody	Thermo Fisher	Cat# 12,380
Rabbit anti‐GFP antibody	Abcam	Cat# ab290; RRID: AB_303395
Mouse anti‐ATP5A antibody		Cat# ab14748; RRID: AB_301447
Rabbit anti‐Phospho‐S6 Kinase Ribosomal Protein (Thr398)	Cell Signaling Technology	Cat# 9,209; RRID: AB_2269804
Mouse anti‐Phospho‐S6 Kinase Ribosomal Protein (Thr389)		Cat# 9,206; RRID: AB_2285392
Rabbit anti‐phosphorylated ribosomal protein S6 antibody	(Kim, Jang, Yang, & Chung, 2017)	N/A
Rabbit anti‐Rab7	(Jung et al., 2017)	N/A
*Drosophila* Strains	Source	Cat#
*w^1118^*	Bloomington stock center (BDSC)	3,605
Rab27^EYFP^		62,556
*elav*‐GS‐Gal4		43,642
*238Y*‐Gal4		81,009
*dilp2‐*Gal4		37,516
*SOG*‐Gal4		37,295
*G0050*‐Gal4		N/A
*R16A06*‐Gal4		48,709
*c708a*‐Gal4		50,743
*N‐syb‐*Gal4		51,635
*tub*‐Gal80^ts^		7,108
UAS*‐YFP‐rab27*		9,810
UAS*‐rab27‐RNAi*		31,887
UAS*‐mCD8‐GFP*		5,137
UAS*‐mCD8‐RFP*		27,398
UAS‐*reaper*		5,824
UAS*‐NaCh*		9,469
UAS‐*NFAT*		66,542
UAS‐*ricin/CyO*		28,998
UAS*‐tsc2*		80,576
UAS*‐s6k^DN^*		6,911
UAS*‐s6k^CA^*		6,914
UAS*‐YFP‐rab27^CA^*		23,266
UAS*‐YFP‐rab6^CA^*		9,776
UAS‐*Denmark*		33,062
UAS‐*thor*		9,147
UAS*‐kaede*		26,161
UAS‐*Denmark,syt‐eGFP*		33,065
*VT49246*‐Gal4	Vienna Drosophila RNAi Center‐ Vienna Tile (VDRC)	205,379
*VT14429*‐Gal4		204,199
*VT24615*‐Gal4		203,429
*rab27^Gal4‐KO^*	(Chan et al., 2011)	
*rab27*‐Gal4		
*rab27^Crispr‐KO^*	This study	
UAS‐*s6*‐S5D		

### 4.2 Fly husbandry and stocks

Flies were maintained with standard cornmeal medium at 25℃, 60% humidity in a 12:12 hr light/dark (LD) cycle. *w^1118^* was used as the WT control for all experiments unless otherwise stated. All of the experimental flies were backcrossed 10 times to the isogenic *w^1118^* to remove possible background mutations, except for (reaper (Figure S6a) and ricin (Figure S6c), both of which have been isogenized for 3 generations. To determine the effect of different genetic backgrounds on lifespan, we outcrossed *rab27KO* flies to Canton‐S wild‐type strain and measured lifespan under starvation. To activate GeneSwitch‐Gal4, 100 µl of 200 µM RU486 (mifepristone, Tokyo Chemical Industry #84371‐65–3) in ethanol was added to the surface of the food. Rapamycin (LC laboratories #R‐5000) was dissolved in ethanol and added to the food to inhibit TOR signaling at a final concentration of 200 µM.

### 4.3 Generation of *rab27* knockout flies using CRISPR/Cas9 system

The *rab27* knockout flies (*rab27^Crispr‐KO^)* were generated utilizing the CRISPR/Cas9 system as described in (Jung et al., 2017) with modifications. One pair of gRNAs was designed to target the start codon and 3’ UTR of *rab27* locus for the removal of the entire coding region. Further details are provided in Supporting Information.

### 4.4 Lifespan analysis and antibiotic treatment of *Wolbachia* infection

Lifespan was measured with two independent methods. The “cage” method was modified from (Bai et al., 2015). Briefly, flies were raised at a density of approximately 200 larvae per bottle. Newly eclosed flies were allowed to mate within 48 hr and then transferred to experimental cages at a density of 100 males and 100 females in a 1‐L cage with good ventilation. Fresh food was provided, and deaths were scored every 2–3 days. For the “vial” method, 10 newly hatched flies of the same sex were reared in a standard fly vial. Flies were transferred to fresh vials and dead flies were removed and scored every 2–3 days. To create starvation‐induced stress, 21‐day‐old adult flies were transferred to vials containing 1% agar. Flies were separated by sex into 10 flies per vial, and dead flies were counted every 6 hr. To assay oxidative stress resistance, flies were exposed to filter paper soaked with 5 mM paraquat (PQ) dissolved in 6.5% sucrose solution at the bottom of vials. The effects on survival were analyzed by the log‐rank test. The results of all lifespan experiments were summarized in Tables S1 and S2. For the antibiotic treatment, the flies were reared for three generations with 50 µg/ml tetracycline (Omics Bio) added to the food (Rottschaefer & Lazzaro, 2012). The infection status for *Wolbachia* was then verified via qRT‐PCR with the *wspB* primers. Further details are provided in Supporting Information.

### 4.5 Quantitative Real‐Time PCR (qRT‐PCR)

qRT‐PCR was performed following the guideline of (Bustin et al., 2019). The procedures were as previously described in (Yang et al., 2017). Further details are provided in Supporting Information.

### 4.6 Measurements of larval length and pupation timing

Body lengths were measured at 24, 48, and 72 hr after egg collection. Larvae were fixed in 4% paraformaldehyde and then washed with phosphate‐buffered saline (PBS). Images of larvae were taken with a Canon EOS‐700D digital camera on a Leica S8APO microscope, and the body lengths were measured with ImageJ. To measure pupation time, eggs were collected for 24 hr and the number of pupae was recorded every 24 hr.

### 4.7 Immunohistochemistry and confocal imaging

Adult fly brains were dissected, immunostained, and imaged following (Jung et al., 2017) with modifications. Further details are provided in Supporting Information.

### 4.8 Quantification of dFOXO staining

A region of interest (ROI) was drawn around the DAPI positive nuclei, and an equally sized circle was drawn to mark the ROI in the cytoplasm in the same image. The green signal (dFOXO staining) was measured in these areas from individual confocal images using ImageJ. The ratio of cytoplasmic‐to‐+nuclear dFOXO was calculated by dividing the mean fluorescent intensity of cytoplasmic dFOXO to the mean fluorescent intensity of nuclear dFOXO from the same image. We then plotted all data in boxplots and used an unpaired Student's *t* test for significant differences.

### 4.9 Triglycerides (TAG) and glucose measurement

TAG measurement was performed according to (Slack et al., 2010) with modifications. Further details are provided in Supporting Information.

### 4.10 Feeding assay

21‐day‐old adult flies were starved for 6 hr. Subsequently, ten single‐sex flies were transferred into a new vial containing standard cornmeal food and blue dye (0.0375 mg/ml, Sigma‐Aldrich #3844‐45–9) for 3 hr. Flies were homogenized with 400 µl PBS. After centrifugation, the amount of food ingested was determined by absorbance at wavelength 620 nm.

### 4.11 Body weight measurement

Ten 21‐day‐old flies were weighed on a microbalance (Denver Instrument TB‐124). Body weight measurements were performed in triplicates for each sex of each strain.

### 4.12 Female fecundity

Eggs laid by mated female flies were counted daily from flies that were 7‐day post‐eclosion for 35 consecutive days. Fresh standard food was changed daily.

### 4.13 Olfactory aversive memory

Conditioned odor avoidance was performed as previously described (Yang et al., 2017). Further details are provided in Supporting Information.

### 4.14 Western blot and co‐immunoprecipitation (co‐IP)

Western blot was performed as previously described (Yang et al., 2017). For co‐IP experiments, 200 adult heads were collected through a small sieve. The head lysate was incubated with protein G agarose beads to minimize nonspecific binding. And the remaining lysate was incubated with GFP antibody‐bound Mag beads (GE healthcare #28‐9670–70) overnight, and then the beads were washed and boiled. Further details are provided in Supporting Information.

### 4.15 Sucrose density gradient fractions

500 adult flies were flash‐frozen in liquid nitrogen, vortexed, and passed through a small sieve to collect fly heads. Adult heads were homogenized using a pestle in lysis buffer (50 mM Tris pH8.0, 150 mM NaCl, 2 mM EDTA, 1% igepal, 0.5% sodium deoxycholate) with protease inhibitor cocktail (Roche). The lysate supernatant was layered on 20%–55% sucrose gradient. The gradient was centrifuged for 16 hr at 35,000 rpm at 4℃ in an SW‐41 or SW‐55 Ti rotor (Beckman). Serial fractions (1 ml each) were collected from the top of the tube and analyzed by Western Blotting. The band intensity was quantified with ImageJ software.

### 4.16 Proximity ligation assay (PLA)

The PLA assay was performed according to (Mosca et al., 2017). Further details are provided in Supporting Information.

### 4.17 Kaede measurement

To measure the amount of newly synthesized Kaede proteins, pre‐existing Kaede was first photoconverted into red fluorescent proteins by UV irradiation. After 6‐hr of UV irradiation, the flies were kept at 25℃, 60% humidity for 60 min. Next, the brains were dissected in PBS and fixed in 4% paraformaldehyde in PBS at room temperature for 45 min. The brains were then washed in 0.5% PBST three times for 20 min and mounted with Vectashield. The images were quantified and measured as described.

### 4.18 Quantification and statistical analysis

Each experiment was performed at least three biological replicates in all graphs. For fluorescence images, the intensity was quantified double‐blindly and measured using Adobe Photoshop CS6 and ImageJ. The band intensity of Western blotting or co‐IP was quantified with ImageJ. All data were expressed as mean ± *SEM* and were compared using ANOVA followed by a Tukey test (for experimental groups ≥ 3) or an unpaired Student's *t* test (for experimental groups = 2). Survival data were analyzed by log‐rank tests (Gronke et al., 2010). All statistical analysis was carried out using GraphPad Prism 5 software. A *p* < .05 was considered statistically significant: * indicates* p* < .05; ** indicates *p* < .01; *** indicates *p* < .001. All images were processed in Adobe Photoshop and assembled with Adobe Illustrator.

## CONFLICT OF INTERESTS

The authors declare no competing interests.

## AUTHOR CONTRIBUTIONS

W.‐Y.L. and C.‐C.C. conceptualized the study; W.‐Y.L., Y.‐T.C., J.‐R.L., and J.‐K.W. involved in methodology; W.‐Y.L., Y.‐T.C., Y.‐J.L., J.‐K.W., K.‐L.H., J.‐R.L., S.‐C.L., C.‐C.H., H.‐D.W., C.‐L.W., S.‐Y.H., and C.‐C.C. investigated the study; W.Y.L., S.‐Y.H., and C.‐C.C. wrote the original draft; W.‐Y.L., Y.‐T.C., Y.‐J.L., J.‐K.W., K.‐L.H., J.‐R.L., S.‐C.L., C.‐C.H., H.‐D.W., C.‐L.W., S.‐Y.H., and C.‐C.C. wrote, reviewed, and edited the article; C.‐C.C. involved in funding acquisition; C.‐C.C. performed supervision.

## Supporting information

Supplementary Material

## Data Availability

The data that support the findings of this study are available from the corresponding author upon reasonable request.

## References

[acel13179-bib-0001] Aso, Y. , Grubel, K. , Busch, S. , Friedrich, A. B. , Siwanowicz, I. , & Tanimoto, H. (2009). The mushroom body of adult Drosophila characterized by GAL4 drivers. Journal of Neurogenetics, 23(1–2), 156–172. 10.1080/01677060802471718 19140035

[acel13179-bib-0002] Bai, H. , Kang, P. , & Tatar, M. (2012). Drosophila insulin‐like peptide‐6 (dilp6) expression from fat body extends lifespan and represses secretion of Drosophila insulin‐like peptide‐2 from the brain. Aging Cell, 11(6), 978–985. 10.1111/acel.12000 22935001 PMC3500397

[acel13179-bib-0003] Bai, H. , Post, S. , Kang, P. , & Tatar, M. (2015). Drosophila longevity assurance conferred by reduced insulin receptor substrate Chico partially requires d4eBP. PLoS One, 10(8), e0134415 10.1371/journal.pone.0134415 26252766 PMC4529185

[acel13179-bib-0004] Bang, S. , Hyun, S. , Hong, S.‐T. , Kang, J. , Jeong, K. , Park, J.‐J. , … Chung, J. (2011). Dopamine signalling in mushroom bodies regulates temperature‐preference behaviour in Drosophila. PLoS Genetics, 7(3), e1001346 10.1371/journal.pgen.1001346 21455291 PMC3063753

[acel13179-bib-0005] Bjedov, I. , Toivonen, J. M. , Kerr, F. , Slack, C. , Jacobson, J. , Foley, A. , & Partridge, L. (2010). Mechanisms of life span extension by rapamycin in the fruit fly Drosophila melanogaster. Cell Metabolism, 11(1), 35–46. 10.1016/j.cmet.2009.11.010 20074526 PMC2824086

[acel13179-bib-0006] Böhni, R. , Riesgo‐Escovar, J. , Oldham, S. , Brogiolo, W. , Stocker, H. , Andruss, B. F. , … Hafen, E. (1999). Autonomous control of cell and organ size by CHICO, a Drosophila homolog of vertebrate IRS1‐4. Cell, 97(7), 865–875. 10.1016/S0092-8674(00)80799-0 10399915

[acel13179-bib-0007] Broughton, S. J. , Piper, M. D. W. , Ikeya, T. , Bass, T. M. , Jacobson, J. , Driege, Y. , … Partridge, L. (2005). Longer lifespan, altered metabolism, and stress resistance in Drosophila from ablation of cells making insulin‐like ligands. Proceedings of the National Academy of Sciences U S A, 102(8), 3105–3110. 10.1073/pnas.0405775102 PMC549445 15708981

[acel13179-bib-0008] Bustin, S. A. , Benes, V. , Garson, J. A. , Hellemans, J. , Huggett, J. , Kubista, M. , … Wittwer, C. T. (2019). The MIQE guidelines: minimum information for publication of quantitative real‐time PCR experiments. Clinical Chemistry, 55(4), 611–622. 10.1373/clinchem.2008.112797 19246619

[acel13179-bib-0009] Chan, C.‐C. , Scoggin, S. , Wang, D. , Cherry, S. , Dembo, T. , Greenberg, B. , … Hiesinger, P. R. (2011). Systematic discovery of Rab GTPases with synaptic functions in Drosophila. Current Biology, 21(20), 1704–1715. 10.1016/j.cub.2011.08.058 22000105 PMC3351199

[acel13179-bib-0010] Demontis, F. , & Perrimon, N. (2010). FOXO/4E‐BP signaling in Drosophila muscles regulates organism‐wide proteostasis during aging. Cell, 143(5), 813–825. 10.1016/j.cell.2010.10.007 21111239 PMC3066043

[acel13179-bib-0011] Dunst, S. , Kazimiers, T. , von Zadow, F. , Jambor, H. , Sagner, A. , Brankatschk, B. , … Brankatschk, M. (2015). Endogenously tagged rab proteins: A resource to study membrane trafficking in Drosophila. Developmental Cell, 33(3), 351–365. 10.1016/j.devcel.2015.03.022 25942626 PMC4431667

[acel13179-bib-0012] Dus, M. , Lai, J.‐Y. , Gunapala, K. M. , Min, S. , Tayler, T. D. , Hergarden, A. C. , … Suh, G. S. B. (2015). Nutrient sensor in the brain directs the action of the brain‐gut axis in Drosophila. Neuron, 87(1), 139–151. 10.1016/j.neuron.2015.05.032 26074004 PMC4697866

[acel13179-bib-0013] Flatt, T. , Min, K.‐J. , D'Alterio, C. , Villa‐Cuesta, E. , Cumbers, J. , Lehmann, R. , … Tatar, M. (2008). Drosophila germ‐line modulation of insulin signaling and lifespan. Proceedings of the National Academy of Sciences U S A, 105(17), 6368–6373. 10.1073/pnas.0709128105 PMC2359818 18434551

[acel13179-bib-0014] Fukuda, M. (2006). Rab27 and its effectors in secretory granule exocytosis: A novel docking machinery composed of a Rab27.effector complex. Biochemical Society Transactions, 34(Pt 5), 691–695. 10.1042/BST0340691 17052176

[acel13179-bib-0015] Geminard, C. , Rulifson, E. J. , & Leopold, P. (2009). Remote control of insulin secretion by fat cells in Drosophila. Cell Metabolism, 10(3), 199–207. 10.1016/j.cmet.2009.08.002 19723496

[acel13179-bib-0017] Gronke, S. , Clarke, D. F. , Broughton, S. , Andrews, T. D. , & Partridge, L. (2010). Molecular evolution and functional characterization of Drosophila insulin‐like peptides. PLoS Genetics, 6(2), e1000857 10.1371/journal.pgen.1000857 20195512 PMC2829060

[acel13179-bib-0018] Hansen, M. , Flatt, T. , & Aguilaniu, H. (2013). Reproduction, fat metabolism, and life span: What is the connection? Cell Metabolism, 17(1), 10–19. 10.1016/j.cmet.2012.12.003 23312280 PMC3567776

[acel13179-bib-0019] Hu, D. , Cao, P. , Thiels, E. , Chu, C. T. , Wu, G. Y. , Oury, T. D. , & Klann, E. (2007). Hippocampal long‐term potentiation, memory, and longevity in mice that overexpress mitochondrial superoxide dismutase. Neurobiology of Learning and Memory, 87(3), 372–384. 10.1016/j.nlm.2006.10.003 17129739 PMC1847321

[acel13179-bib-0020] Hwangbo, D. S. , Gershman, B. , Tu, M. P. , Palmer, M. , & Tatar, M. (2004). Drosophila dFOXO controls lifespan and regulates insulin signalling in brain and fat body. Nature, 429(6991), 562–566. 10.1038/nature02549 15175753

[acel13179-bib-0021] Jin, E. J. , Chan, C. C. , Agi, E. , Cherry, S. , Hanacik, E. , Buszczak, M. , & Hiesinger, P. R. (2012). Similarities of Drosophila rab GTPases based on expression profiling: Completion and analysis of the rab‐Gal4 kit. PLoS One, 7(7), e40912 10.1371/journal.pone.0040912 22844416 PMC3402473

[acel13179-bib-0022] Joiner, W. J. , Crocker, A. , White, B. H. , & Sehgal, A. (2006). Sleep in Drosophila is regulated by adult mushroom bodies. Nature, 441(7094), 757–760. 10.1038/nature04811 16760980

[acel13179-bib-0023] Jung, W. H. , Liu, C. C. , Yu, Y. L. , Chang, Y. C. , Lien, W. Y. , Chao, H. C. , … Chan, C. C. (2017). Lipophagy prevents activity‐dependent neurodegeneration due to dihydroceramide accumulation in vivo. EMBO Reports, 18(7), 1150–1165. 10.15252/embr.201643480 28507162 PMC5494533

[acel13179-bib-0024] Kapahi, P. , Zid, B. M. , Harper, T. , Koslover, D. , Sapin, V. , & Benzer, S. (2004). Regulation of lifespan in Drosophila by modulation of genes in the TOR signaling pathway. Current Biology, 14(10), 885–890. 10.1016/j.cub.2004.03.059 15186745 PMC2754830

[acel13179-bib-0025] Kaplan, B. , Qazi, Y. , & Wellen, J. R. (2014). Strategies for the management of adverse events associated with mTOR inhibitors. Transplantation Reviews (Orlando), 28(3), 126–133. 10.1016/j.trre.2014.03.002 24685370

[acel13179-bib-0026] Kasai, K. , Ohara‐Imaizumi, M. , Takahashi, N. , Mizutani, S. , Zhao, S. , Kikuta, T. , … Izumi, T. (2005). Rab27a mediates the tight docking of insulin granules onto the plasma membrane during glucose stimulation. Journal of Clinical Investigation, 115(2), 388–396. 10.1172/JCI22955 15690086 PMC546426

[acel13179-bib-0027] Kim, W. , Jang, Y.‐G. , Yang, J. , & Chung, J. (2017). Spatial activation of TORC1 is regulated by hedgehog and E2F1 signaling in the Drosophila eye. Developmental Cell, 42(4), 363–375.e364. 10.1016/j.devcel.2017.07.020 28829944

[acel13179-bib-0028] Leal, G. , Comprido, D. , & Duarte, C. B. (2014). BDNF‐induced local protein synthesis and synaptic plasticity. Neuropharmacology, 76, 639–656. 10.1016/j.neuropharm.2013.04.005 23602987

[acel13179-bib-0029] Libert, S. , Zwiener, J. , Chu, X. , Vanvoorhies, W. , Roman, G. , & Pletcher, S. D. (2007). Regulation of Drosophila life span by olfaction and food‐derived odors. Science, 315(5815), 1133–1137. 10.1126/science.1136610 17272684

[acel13179-bib-0030] Mahoney, T. R. , Liu, Q. , Itoh, T. , Luo, S. , Hadwiger, G. , Vincent, R. , … Nonet, M. L. (2006). Regulation of synaptic transmission by RAB‐3 and RAB‐27 in *Caenorhabditis elegans* . Molecular Biology of the Cell, 17(6), 2617–2625. 10.1091/mbc.e05-12-1170 16571673 PMC1474797

[acel13179-bib-0031] Maklakov, A. A. , & Immler, S. (2016). The expensive germline and the evolution of ageing. Current Biology, 26(13), R577–R586. 10.1016/j.cub.2016.04.012 27404253

[acel13179-bib-0032] Martins, R. , Lithgow, G. J. , & Link, W. (2016). Long live FOXO: Unraveling the role of FOXO proteins in aging and longevity. Aging Cell, 15(2), 196–207. 10.1111/acel.12427 26643314 PMC4783344

[acel13179-bib-0033] Masuyama, K. , Zhang, Y. , Rao, Y. , & Wang, J. W. (2012). Mapping neural circuits with activity‐dependent nuclear import of a transcription factor. Journal of Neurogenetics, 26(1), 89–102. 10.3109/01677063.2011.642910 22236090 PMC3357894

[acel13179-bib-0034] Mattison, J. A. , Colman, R. J. , Beasley, T. M. , Allison, D. B. , Kemnitz, J. W. , Roth, G. S. , … Anderson, R. M. (2017). Caloric restriction improves health and survival of rhesus monkeys. Nature Communications, 8, 14063 10.1038/ncomms14063 PMC5247583 28094793

[acel13179-bib-0035] McBride, S. M. J. , Choi, C. H. , Wang, Y. , Liebelt, D. , Braunstein, E. , Ferreiro, D. , … Jongens, T. A. (2005). Pharmacological rescue of synaptic plasticity, courtship behavior, and mushroom body defects in a Drosophila model of fragile X syndrome. Neuron, 45(5), 753–764. 10.1016/j.neuron.2005.01.038 15748850

[acel13179-bib-0036] Min, K.‐T. , & Benzer, S. (1997). Wolbachia, normally a symbiont of Drosophila, can be virulent, causing degeneration and early death. Proceedings of the National Academy of Sciences USA, 94(20), 10792–10796. 10.1073/pnas.94.20.10792 PMC23488 9380712

[acel13179-bib-0037] Mosca, T. J. , Luginbuhl, D. J. , Wang, I. E. , & Luo, L. (2017). Presynaptic LRP4 promotes synapse number and function of excitatory CNS neurons. eLife, 6, e27347 10.7554/eLife.27347 28606304 PMC5469616

[acel13179-bib-0038] Partridge, L. , Gems, D. , & Withers, D. J. (2005). Sex and death: What is the connection? Cell, 120(4), 461–472. 10.1016/j.cell.2005.01.026 15734679

[acel13179-bib-0039] Rottschaefer, S. M. , & Lazzaro, B. P. (2012). No effect of wolbachia on resistance to intracellular infection by pathogenic bacteria in *Drosophila melanogaster* . PLoS One, 7(7), e40500 10.1371/journal.pone.0040500 22808174 PMC3394738

[acel13179-bib-0040] Rulifson, E. J. , Kim, S. K. , & Nusse, R. (2002). Ablation of insulin‐producing neurons in flies: Growth and diabetic phenotypes. Science, 296(5570), 1118–1120. 10.1126/science.1070058 12004130

[acel13179-bib-0041] Satoh, A. , Brace, C. S. , Rensing, N. , Cliften, P. , Wozniak, D. F. , Herzog, E. D. , … Imai, S.‐I. (2013). Sirt1 extends life span and delays aging in mice through the regulation of Nk2 homeobox 1 in the DMH and LH. Cell Metabolism, 18(3), 416–430. 10.1016/j.cmet.2013.07.013 24011076 PMC3794712

[acel13179-bib-0042] Slack, C. , Alic, N. , Foley, A. , Cabecinha, M. , Hoddinott, M. P. , & Partridge, L. (2015). The Ras‐Erk‐ETS‐Signaling pathway is a drug target for longevity. Cell, 162(1), 72–83. 10.1016/j.cell.2015.06.023 26119340 PMC4518474

[acel13179-bib-0043] Slack, C. , Giannakou, M. E. , Foley, A. , Goss, M. , & Partridge, L. (2011). dFOXO‐independent effects of reduced insulin‐like signaling in Drosophila. Aging Cell, 10(5), 735–748. 10.1111/j.1474-9726.2011.00707.x 21443682 PMC3193374

[acel13179-bib-0044] Slack, C. , Werz, C. , Wieser, D. , Alic, N. , Foley, A. , Stocker, H. , … Partridge, L. (2010). Regulation of lifespan, metabolism, and stress responses by the Drosophila SH2B protein, Lnk. Plos Genetics, 6(3), e1000881 10.1371/journal.pgen.1000881 20333234 PMC2841611

[acel13179-bib-0045] Tsao, C. H. , Chen, C. C. , Lin, C. H. , Yang, H. Y. , & Lin, S. (2018). Drosophila mushroom bodies integrate hunger and satiety signals to control innate food‐seeking behavior. eLife, 7, e35264 10.7554/eLife.35264 29547121 PMC5910021

[acel13179-bib-0046] Tsuboi, T. , & Fukuda, M. (2006). Rab3A and Rab27A cooperatively regulate the docking step of dense‐core vesicle exocytosis in PC12 cells. Journal of Cell Science, 119(Pt 11), 2196–2203. 10.1242/jcs.02962 16684812

[acel13179-bib-0047] Yang, C. N. , Wu, M. F. , Liu, C. C. , Jung, W. H. , Chang, Y. C. , Lee, W. P. , … Chan, C. C. (2017). Differential protective effects of connective tissue growth factor against Abeta neurotoxicity on neurons and glia. Human Molecular Genetics, 26(20), 3909–3921. 10.1093/hmg/ddx278 29016849

[acel13179-bib-0048] Yang, S. B. , Tien, A. C. , Boddupalli, G. , Xu, A. W. , Jan, Y. N. , & Jan, L. Y. (2012). Rapamycin ameliorates age‐dependent obesity associated with increased mTOR signaling in hypothalamic POMC neurons. Neuron, 75(3), 425–436. 10.1016/j.neuron.2012.03.043 22884327 PMC3467009

[acel13179-bib-0049] Yu, E. , Kanno, E. , Choi, S. , Sugimori, M. , Moreira, J. E. , Llinas, R. R. , & Fukuda, M. (2008). Role of Rab27 in synaptic transmission at the squid giant synapse. Proceedings of the National Academy of Sciences U S A, 105(41), 16003–16008. 10.1073/pnas.0804825105 PMC2562534 18840683

[acel13179-bib-0050] Zhang, G. , Li, J. , Purkayastha, S. , Tang, Y. , Zhang, H. , Yin, Y. , … Cai, D. (2013). Hypothalamic programming of systemic ageing involving IKK‐beta, NF‐kappaB and GnRH. Nature, 497(7448), 211–216. 10.1038/nature12143 23636330 PMC3756938

[acel13179-bib-0051] Zhang, Y. , Lanjuin, A. , Chowdhury, S. R. , Mistry, M. , Silva‐García, C. G. , Weir, H. J. , … Mair, W. B. (2019). Neuronal TORC1 modulates longevity via AMPK and cell nonautonomous regulation of mitochondrial dynamics in *C. elegans* . eLife, 8, e49158 10.7554/eLife.49158 31411562 PMC6713509

[acel13179-bib-0052] Zhao, X. L. , & Campos, A. R. (2012). Insulin signalling in mushroom body neurons regulates feeding behaviour in Drosophila larvae. Journal of Experimental Biology, 215(Pt 15), 2696–2702. 10.1242/jeb.066969 22786647

[acel13179-bib-0053] Zhao, Z.‐D. , Yang, W. Z. , Gao, C. , Fu, X. , Zhang, W. , Zhou, Q. , … Shen, W. L. (2017). A hypothalamic circuit that controls body temperature. Proceedings of the National Academy of Sciences U S A, 114(8), 2042–2047. 10.1073/pnas.1616255114 PMC5338448 28053227

[acel13179-bib-0054] Zullo, J. M. , Drake, D. , Aron, L. , O’Hern, P. , Dhamne, S. C. , Davidsohn, N. , … Yankner, B. A. (2019). Regulation of lifespan by neural excitation and REST. Nature, 574(7778), 359–364. 10.1038/s41586-019-1647-8 31619788 PMC6893853

