# Supplementary material for: Lifespan regulation in α/β posterior neurons of the fly mushroom bodies by Rab27
Source: Aging Cell. 2020 Jul 6;19(8):e13179. doi: 10.1111/acel.13179 (PMC7431830; doi:10.1111/acel.13179)
Supplement: Supplementary file 1 — Supplementary Material [file ACEL-19-e13179-s001.docx]

**SUPPORTING INFORMATION**

**Lifespan regulation in α/β posterior neurons of the fly mushroom bodies by Rab27**

Wen-Yu Lien, Yu-Ting Chen, Yi-Jhan Li, Jie-Kai Wu, Kuan-Lin Huang, Jian-Rong Lin, Shih-Ching Lin, Chia-Chun Hou, Horng-Dar Wang, Chia-Lin Wu, Shu-Yi Huang, Chih-Chiang Chan

**Supplementary Materials**

**Generation of *rab27* knockout flies using CRISPR/Cas9 system**

The *rab27* knockout flies (*rab27^Crispr-KO^)* were generated utilizing the CRISPR/Cas9 system as described in ([Jung et al., 2017](#_ENREF_23)) with modifications. Briefly, one pair of gRNAs was designed to target the start codon and 3’ UTR of *rab27* locus for the removal of the entire coding region. The following gRNA primers were used:

ATG-*rab27*-fwd: CTTCCCTCTGCAATTAGCCGGATC

ATG-*rab27*-rev: AAACGATCCGGCTAATTGCAGAGG

3’UTR-*rab27*-fwd: CTTCGATAACTGATAGCTGCGGAA

3’UTR-*rab27*-rev: AAACTTCCGCAGCTATCAGTTATC

The gRNA pair was cloned into pBFv-U6.2B, and the construct was injected into the embryos of P{nos-phiC31\int.NLS}X; P{CaryP}attP40 flies crossed to P{nos-Cas9, y+, v+} flies for germline-specific deletion of *rab27*. Candidate *rab27^Crispr-KO^* flies were identified by a PCR screen and confirmed by sequencing.

The primer set for PCR screen is shown below:

Screen-*rab27*-fwd: GAAAGCTGGCGCAAGCTTTGG

Screen-*rab27*-rev: CGTGGAGTACTACCGCCAGTG

**Quantitative Real-Time PCR (qRT-PCR)**

To confirm the knockdown efficiency of *rab27*, total RNA was prepared from 50 heads using the NucleoSpin RNA Kit (Macherey-Nagel). Reverse transcription was performed using an Oligo (dT)_18_ primer (Invitrogen) and SuperScript III Reverse Transcriptase (Invitrogen, USA). qRT-PCR was performed with IQ2 SYBR Green Fast qPCR System Master Mix (Bio Genesis) using a 7300 Real-Time PCR System (Applied Biosystems). Each sample was run in triplicates and the average value for each sample was normalized to *rp49*. Each result represents three independent experiments, and the error bar denotes the standard deviation of the mean (SEM). The primers used are listed below:

*rab27*-fwd: 5'-AGGAGCGCTTCCGTTCACTA-3'

*rab27*-rev: 5'-GCCGTCTCCAGGAAGCTCTT-3'

*rp49*-fwd: 5'-ACTTCATCCGCCACCAGTCG-3'

*rp49*-rev: 5'-CGGGTGCGCTTGTTCGATCC-3'

To confirm the status of *Wolbachia*, each sample was run in triplicates and the average value for each sample was normalized to *rpl11* ([Grobler et al., 2018](#_ENREF_16)). The primers used are listed below:

*wspB*-fwd: 5'-ACAACAGCTATAGGGCTGAATTGGAA-3'

*wspB*-rev: 5'-TCAGGATCCTCACCAGTCTCCTTTAG-3'

*rpl11*-fwd: 5'-CGAGGGATACCTGTGAGCAGCTT-3'

*rpl11*-rev: 5'-GTCACTTCTTGTGCTGCCATCGT-3'

**Immunohistochemistry and confocal imaging**

Adult fly brains were dissected in PBS, ﬁxed in 4% paraformaldehyde at room temperature for 65 min, and degassed in 2% Triton X-100 in PBS (2% PBST) with 1% bovine serum albumin (BSA, BioShop Canada Inc. #ALB001) for 1 hr. The brains were blocked in fresh 2% PBST with 1% BSA at room temperature for 2 hr. Next, the brains were incubated in primary antibodies diluted in 0.25% PBST with 1% BSA at 4℃ for 1 day, washed with 1% PBST, and incubated in secondary antibodies diluted in 0.25% PBST with 1% BSA at 4℃ for 1 day. The brains were then washed with 1% PBST and mounted in Vectashield (H-1000, Vector Laboratories). Adult fly guts were dissected in PBS, ﬁxed in 4% paraformaldehyde at room temperature for 45 min, and blocked in 0.25% PBST with 1% BSA at room temperature for 1 hr. Next, the guts were incubated in AlexaFluor568 Phalloidin (Thermo Fisher Scientific, #A12380) diluted in 0.25% PBST with 1% BSA at 4℃ for 1 day. The guts were then washed with 0.25% PBST and mounted in Vectashield (H-1200, Vector Laboratories). The images were captured with a Leica TCS SP5 confocal laser scanning microscope (Leica Microsystems, Singapore). Primary antibodies included: Rabbit polyclonal anti-dFOXO (1:1000, Cosmo Bio Co #CAC-THU-A-DFOXO), Mouse monoclonal anti-DLG (1:50, Developmental Studies Hybridoma Bank (DSHB) #4F3), Phalloidin-conjugated with Alexa Fluor-568 (Thermo Fisher Scientific #A12380), Rabbit polyclonal anti-phospho-S6K (1:1000, Cell Signaling #9209), Rabbit polyclonal anti-phospho-S6 (1:400, ([Kim et al., 2017](#_ENREF_27))), Rabbit polyclonal anti-GFP (1:1000, Abcam #ab290). DyLight 405-, Alexa 488-, Cy3-, or Alexa 647-conjugated secondary antibodies (Jackson ImmunoResearch Laboratories) were used at 1:500.

**Triglycerides (TAG) and glucose measurement**

TAG measurement was performed according to ([C. Slack et al., 2010](#_ENREF_44)) with modifications. Briefly, for each trial, 21-day-old adult flies were separated according to the sex and 10 flies in each vial were homogenized in lysis buffer. The lysate was centrifuged at 400 g for 10 min. TAG was measured by mixing 2.5 µL of the supernatant with Triglycerides Reagent (Fortress diagnostics #BXC0271C), incubated at 25℃ for 15 min, and then measured the absorbance at wavelength 490 nm. The measurements were normalized to body weight. Glucose was measured according to ([Broughton et al., 2005](#_ENREF_7); [Rulifson et al., 2002](#_ENREF_40)) with modifications. Briefly, for each trial, hemolymph was collected from 35 ﬂies. One µL of hemolymph was diluted in PBS, mixed with Glucose Reagent (Randox #GL1611), incubated at 37℃ for 5 min, and the absorbance was measured at wavelength 340 nm.

**Olfactory aversive memory**

Groups of ∼100 flies received one training session, during which they were exposed sequentially to one odor (conditioned stimulus, CS+; 3-octanol or 4-methyl-cyclohexanol) paired with electric shock (12 × 1.5-s pulses of 75-V DC electric shock at 5-s interpulse intervals) and then a second odor (CS-; 4-methyl-cyclohexanol or 3-octanol) without electric shock. Conditioned odor avoidance was tested immediately after training. During testing, flies were exposed simultaneously to the CS+ and CS- odors in a T-maze for 2 min. Then, flies were trapped in either T-maze arm, anesthetized, and counted. From this distribution, a performance index was calculated as the number of flies avoiding the shocked odor minus that avoiding the non-shocked odor divided by the total number of flies and multiplied by 100.

**Western blot and co-immunoprecipitation (co-IP)**

Adult fly brains were dissected in PBS and homogenized in lysis buffer (50 mM Tris pH8.0, 150 mM NaCl, 2 mM EDTA, 1% NP40, 0.5% Sodium Deoxycholate) supplemented with protease inhibitor cocktail (Roche). The supernatant was boiled in sample buffer (20% glycerol, 4% SDS, 100 mM Tris pH 6.8, 0.002% Bromophenol blue) at 95℃ for 10 min. After a brief spin down to exclude debris, 12 µL of the protein extracts were electrophoresed on 10% SDS-PAGE gels and transferred to nitrocellulose membranes at 10 V for 90 min. Subsequently, the membranes were blocked in TBS with 0.1% Tween 20 (TBST) containing 5% milk or 5% BSA at room temperature for 1 hr. The membranes were incubated overnight at 4℃ with primary antibodies in TBST. The dilutions of antibodies were as follows: anti-phospho-S6K (1:1000, Cell Signaling #9209), anti-Rab7 (1:1000, Jung et al. 2017), anti-ATP5A (1:1000, Abcam #14748). After washing the blots three times in TBST for 20 min each, blots were incubated with horseradish peroxidase (HRP)-conjugated secondary antibodies (1:5000, Jackson ImmunoResearch Laboratories) in TBST at room temperature for 1 hr. After washing three times in TBST for 20 min, the antibody reactivity was visualized with ECL Western Blotting detection reagents (Pierce) and signals were captured using (BioSpectrum^TM^ 600 Imaging System, UVP Ltd.). For co-IP experiments, Mag beads (GE healthcare #28-9670-70) were incubated with anti-GFP (1:100, DSHB #12A06) overnight. 200 adult flies were flash-frozen in liquid nitrogen, vortexed, and passed through a small sieve to allow the separation of fly heads from the bodies. The adult heads were homogenized using a pestle in lysis buffer (5 mM HEPES, 100 mM NaCl, 5 mM EDTA, 0.5% Igepal (Sigma #I3021) and protease inhibitor cocktail) and shook at 4℃ for 1 hr. All subsequent steps were performed at 4℃. After spin down to exclude debris, the supernatant was incubated with protein G agarose beads for 45 min to minimize nonspecific binding. Subsequently, 30 µL of lysate supernatant was taken as input, and the remaining lysate was incubated with GFP-bound Mag beads overnight, and then the beads were washed with wash buffer (5 mM HEPES, 50 mM NaCl, 5 mM EDTA) and boiled at 100℃ for 10 min. Western blotting was performed according to standard protocols described above using primary antibodies anti-GFP (1:3000, Abcam #ab290) and anti-phospho-S6K (1:1000, Cell Signaling #9209). Rabbit HRP-conjugated secondary antibodies were used at 1:5000.

**4.16 Proximity Ligation Assay (PLA)**

The PLA assay was conducted using the DuoLink Mouse Rabbit in situ PLA kit (Sigma-Aldrich #DUO92101) following the manufacturer’s protocol. After degassing to expel tracheal air, the brains were incubated in the DuoLink blocking buffer at 37℃ for 1 hr and then incubated with primary antibodies diluted in Duolink II Antibody Diluent at 4℃ for 1 day, washed with 1% PBST. Next, the brains were incubated in the anti-mouse and anti-rabbit PLA probes at a 1:5 dilution for 2 hr at 37℃. Next, the brains were washed thrice for 10 min each with 1% PBST, and incubated in Ligation solution for 1 hr at 37℃. The brains were then washed thrice in 1% PBST for 10 min each, incubated in Amplification solution for 2 hr at 37℃, washed thrice for 10 min each in 1% PBST, and incubated in secondary antibodies diluted in 0.25% PBST with 1% BSA at 4℃ for 1 day. The brains were then washed with 1% PBST and mounted in Vectashield. The images were quantified and measured as described.

**LEGENDS OF SUPPLEMENTARY FIGURES
Figure S1. The generation and verification of *rab27^Crispr-KO^* fly, and the confirmation that *rab27KO* alleles extend lifespan**

(a) The schematic view of the *rab27* knock-out strategy using CRISPR/cas9. Two PAM sites flanking the *rab27* locus were targeted by Cas9, resulting in the complete removal of the *rab27* open reading frame (ORF). Arrows indicate the PCR primer pair for detecting the excision. (b) PCR results confirming the deletion of *rab27*-ORF in the *rab27^Crispr-KO^* fly. (c) Results of the single-sex vial assay showing survival of females from *w^1118^* control (black) and two independent *rab27* nulls, *rab27^Crispr-KO^* (green), and *rab27KO* (red), *p* < 0.001, log-rank tests.

**Figure S2. The verification for RU486 application**

(a-d’’) Representative confocal images of (a-a”) elav-GS-Gal4 and (b-b”) UAS-mCD8-GFP fed with solvent as control; (c-d”) elav-GS-Gal4>UAS-mCD8-GFP (green) without (c-c”) or with (d-d”) RU486-mediated transgene induction. Nuclei are labeled by DAPI (blue). Scale bars: 75 µm. (e) Relative abundance of *rab27* mRNA in the heads of *elav*-GS-Gal4>UAS-*rab27*-RNAi flies, *elav*-GS-Gal4/+ flies and UAS-*rab27*-RNAi/+ flies, fed with RU486 normalized to the solvent-fed control (EtOH). *p* < 0.001, one-way ANOVA. (f) Survival of female strains described in (e), *p* < 0.001, log-rank tests.

**Figure S3. The effects of genetic background and *Wolbachia* on the lifespan of *rab27KO***

(a) Survival of females from Canton-S control (black) and *rab27KO* (red) in a Canton-S genetic background under starvation (*p* < 0.001). (b) Relative abundance of *Wolbachia* DNA in strains used in this study. *p* < 0.001, one-way ANOVA. (c) Survival of females from *w^1118^* control (black) and *rab27KO* for tetracycline treatment (red) under starvation (*p* < 0.001). All survival data were analyzed by log-rank tests.

**Figure S4. The expression patterns of *rab27*-Gal4 and relevant Gal4 lines**

(a-a’’’) Representative confocal ﬂuorescence images of rab27-Gal4>UAS-mCD8-GFP (green) which labels *rab27*-expressing cells in the brain, with anti-Brp (nc82, red) which labels the whole brain; DAPI staining, blue. Scale bar, 75 μm. (b-b’’’) Whole-mount imaging of the gut with rab27-Gal4>UAS-mCD8-GFP (green) and phalloidin which labels F-actin (red); DAPI staining, blue. No *rab27* expression (*rab27*-Gal4>UAS-*mCD8*-*GFP*, green) was detected. Scale bar, 250 μm. (c-c”) Whole-mount imaging of the ovary with rab27-Gal4>UAS-mCD8-GFP (green). DAPI staining, blue. No *rab27* expression (*rab27*-Gal4>UAS-*mCD8*-*GFP*, green) was detected. Scale bar, 50 μm. (d) The illustration depicts the major regions discussed in this paper, including the MB, mNSC, and SOG. Inset, magniﬁed image of α/βp neurons in the MB. Abbreviations in parentheses indicate the specific region of expression: mNSC, median neurosecretory cluster; SOG, subesophageal ganglion; P, posterior. (e) Summary of UAS-*mCD8-GFP* expression driven by the indicated Gal4 lines that were used to determine the requirement of *rab27* in lifespan regulation shown in Figure 3. O indicates a positive GFP signal. P, posterior.

**Figure S5. Expression pattern of region-specific Gal4 lines and survival curves of *rab27* knockdown with these lines**

(a-d) Expression patterns of Gal4 lines in the brain visualized by UAS-*mCD8-GFP* (green). The brain was stained with anti-DLG to label general neuropils (red). Scale bars: 75 µm. (a’-d’) Survival of females from *rab27* knockdown strains (red) compared with the corresponding Gal4 (black) and UAS (blue) lines, including *rab27* knockdown in (a’) *rab27*-expressing cells (*rab27*>*rab27*-RNAi), *p* < 0.001; (b’) SOG neurons (*SOG*>*rab27*-RNAi), *p* < 0.001; (c’) α’/β’ lobes of the MB (*G0050*>*rab27*-RNAi); and (d’) γ lobe of the MB (*R16A06*>*rab27*-RNAi), ***p* < 0.01, ****p* < 0.001. All survival data were analyzed by log-rank tests.

**Figure S6. The cell counting of *VT14429*-Gal4 expressing neurons and the characterization of subcellular localization of endogenous Rab27^EYFP^**

(a) Representative confocal ﬂuorescence images of *VT14429*-Gal4 expressing neurons labeled with UAS-*mCD8-GFP* (green) and nuclei marked with DAPI (blue) in a whole-mount adult brain. Measurements represent the mean of five brains. Data are represented as mean ± SEM. Scale bar: 10 µm. (b) Quantification of DAPI and GFP positive (+) cells in *VT14429*-Gal4 expressing neurons; mean ± SEM of three independent experiments. (c-d’’) Representative images of endogenous Rab27^EYFP^ (green) with *VT14429*-Gal4 driving UAS-*mCD8-RFP* (red). (c-c’’) Axons from the α/βp neurons. Scale bar: 12.5 µm. (d-d’’) Cell bodies of the α/βp neurons. Scale bar: 2 µm.

**Figure S7. The α/βp neurons are essential during development and are required for adult lifespan maintenance**

(a) Survival of female flies expressing the pro-apoptotic gene *reaper* in α/βp neurons of the MB (*VT14429*>*reaper*) and controls (UAS*-reaper*/+ and *VT14429*-Gal4/+), *p* < 0.001. (b) Expression of *ricin* with several Gal4 lines caused developmental lethality. * indicates one escaper among ~500 progeny. (c) Survival of female flies expressing the cytotoxic protein Ricin at 29℃ from *tub*-Gal80^ts^;*VT14429*>*ricin* and controls (*tub*-Gal80^ts^;*VT14429*-Gal4/+ and UAS-*ricin*/+), *p* < 0.001. All survival data were analyzed by log-rank tests.

**Figure S8. Expression of *thor* in the α/βp neurons does not alter the longevity**

Survival of females from *VT14429*>*thor* and controls (*VT14429*-Gal4/+ and UAS-*thor*/+), *p* < 0.001, log-rank tests.

.

**Figure S9. Rab27 is detected in the same fractions with p-S6K**

Fractionation of homogenates from adult fly heads expressing Rab27^EYFP^ was immunoblotted with anti-GFP and anti-p-S6K, peaks in fractions 3-5. Fraction number from top to bottom of the tube.

**Figure S10. Rab27 anchors S6K in the dendrites and the axons of the α/βp neurons**

(a-b’’’) Representative confocal ﬂuorescence images of the dendrites of the α/βp neurons expressing UAS-*mCD8-GFP* (green) stained with anti-p-S6K (red) and DLG (post-synaptic marker, gray) in *VT14429-*Gal4>UAS-*mCD8-GFP* (a-a’’’) or *rab27 ^Crispr-KO^*;*VT14429*-Gal4>UAS-*mCD8-GFP* (b-b’’’). Scale bars: 10 µm. (c) Quantification of p-S6K intensity in the dendrites by marking DLG positive areas and measured the red signal. The ﬂuorescence intensity was normalized to the control levels. Data are represented as mean ± SEM measured in at least three brains, *p* < 0.05, Student’s t-test. (d-e’’) Representative confocal ﬂuorescence image of the axons of the α/βp neurons expressing UAS-*mCD8-GFP* (green) and stained with anti-p-S6K (red) in *VT14429-*Gal4>UAS-*syt-eGFP* (a-a’’) or *rab27^Crispr-KO^*; *VT14429*-Gal4>UAS-*syt-eGFP* (b-b’’). Scale bars: 5 µm. (f) Quantification of p-S6K intensity in the axons by marking green areas and measured the red signal. The ﬂuorescence intensity was normalized to the control levels. Data are represented as mean ± SEM measured in at least three brains, *p* < 0.05, Student’s t-test.

**Figure S11. Controls of *de novo* protein synthesis in the α/βp neurons with photoconvertible Kaede protein**

(a-b’’’) Adult *VT14429-*Gal4>UAS-*Kaede* or *rab27^Crispr-KO^*;*VT14429*-Gal4>UAS-*Kaede* animals were unexposed to ultraviolet light (UV). Scale bars: 25 µm.

**Figure S12. Expression of the phospho mimetic *s6*-S5D in the α/βp neurons reverses *rab27*-dependent lifespan extension**

(a) Survival of females from *rab27KO* expressing *s6k^CA^* compared with controls (*w^1118^* and *rab27KO*), *p* < 0.001. (b) Survival of the rescue experiment female strains (*rab27^Crispr-KO^*;*VT14429*-Gal4;UAS-*rab27* and *rab27^Crispr-KO^*;*VT14429*-Gal4;UAS-*s6*-S5D), *rab27^Crispr-KO^*;*VT14429*-Gal4, and female controls (*VT14429*-Gal4, UAS-*rab27* and UAS-*s6*-S5D) under starvation, *p* < 0.001. (c) Survival of females from *VT14429*>*rab27*, *VT14429*>*s6*-S5D and controls (*VT14429*-Gal4/+, UAS-*rab27*/+ and UAS-*s6*-S5D/+) under starvation, *p* < 0.001.

All survival data were analyzed by log-rank tests.

**Figure S1**

**
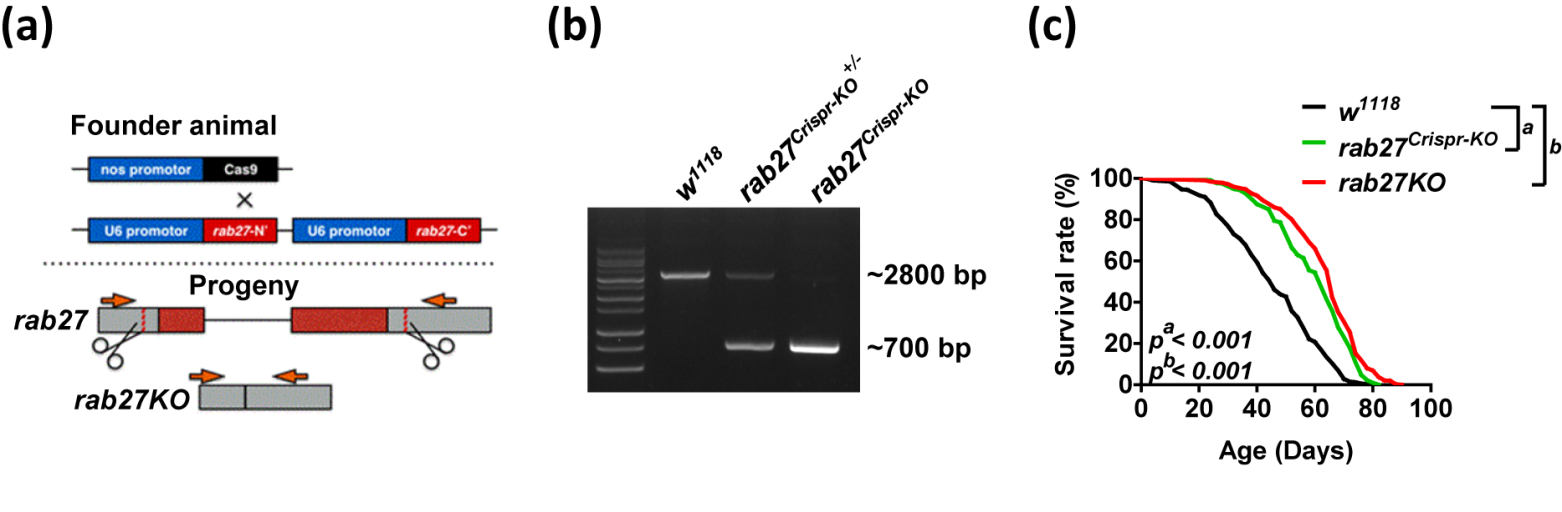
**

**Figure S2**

**
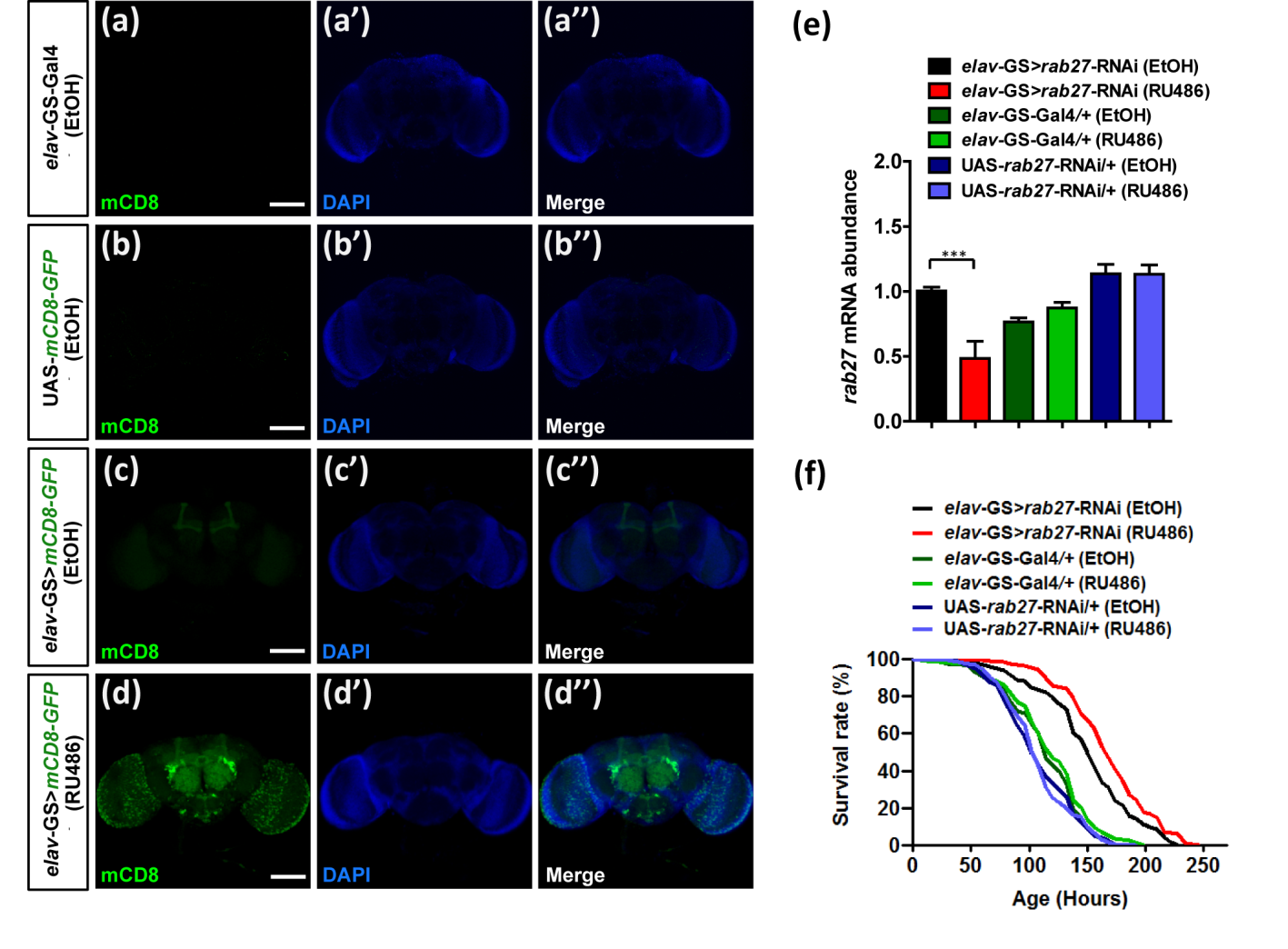
**

**
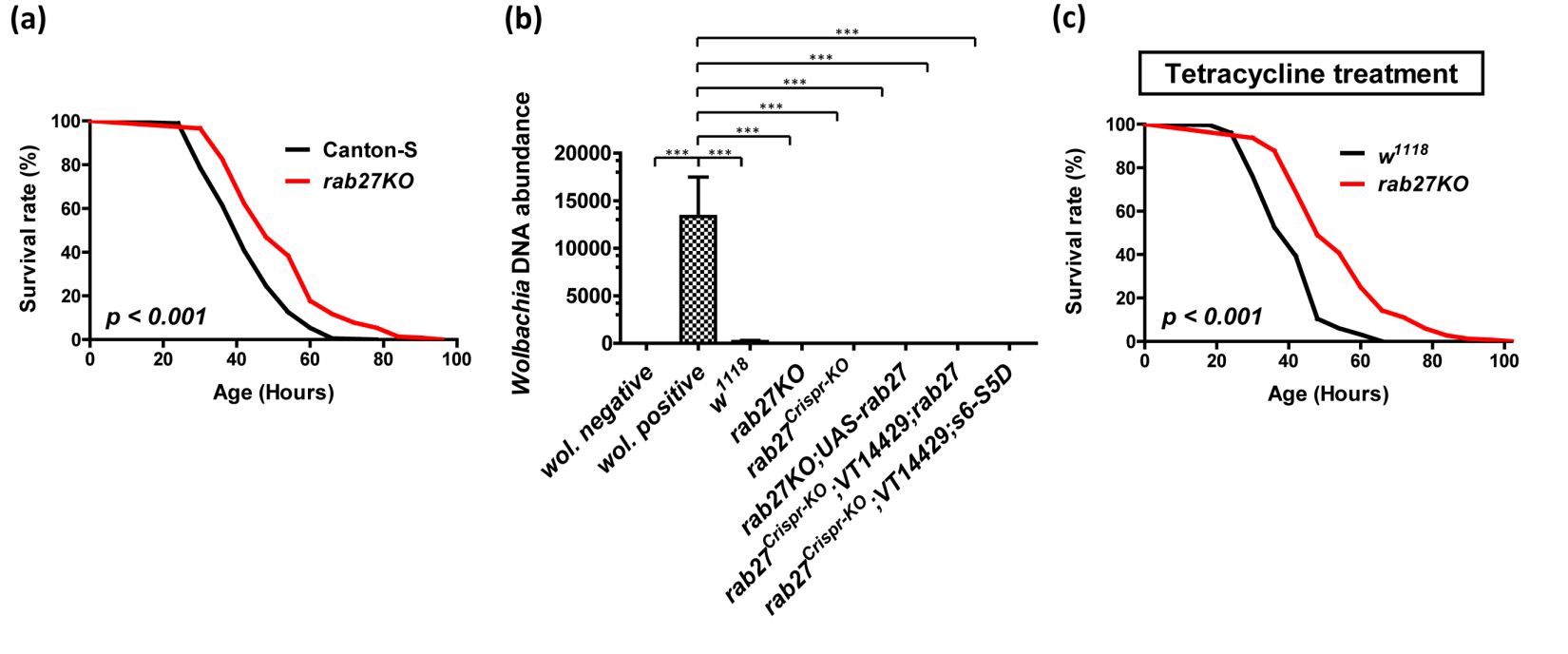
Figure S3**

**Figure S4**

**
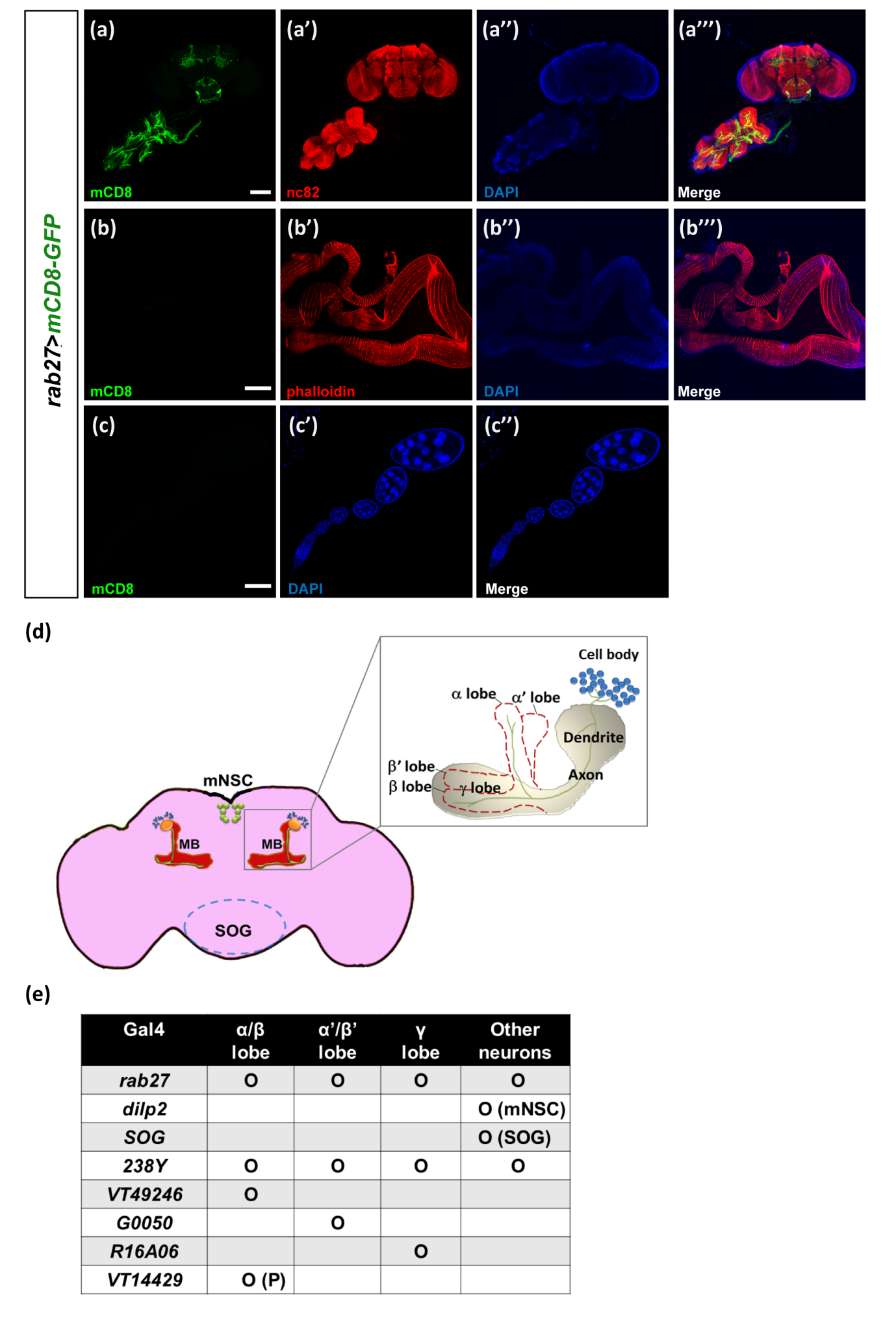
**

**Figure S5**

**
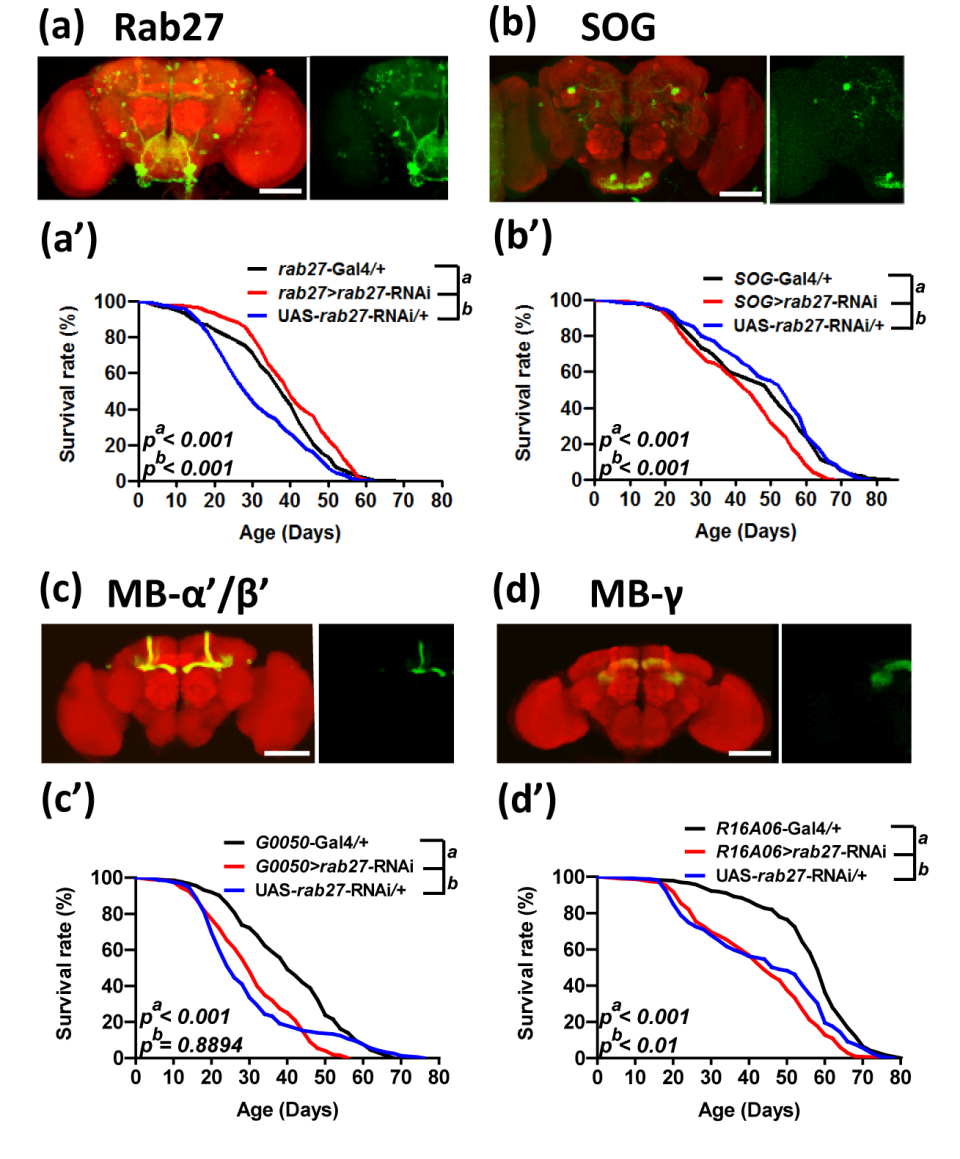
**

**Figure S6**

**
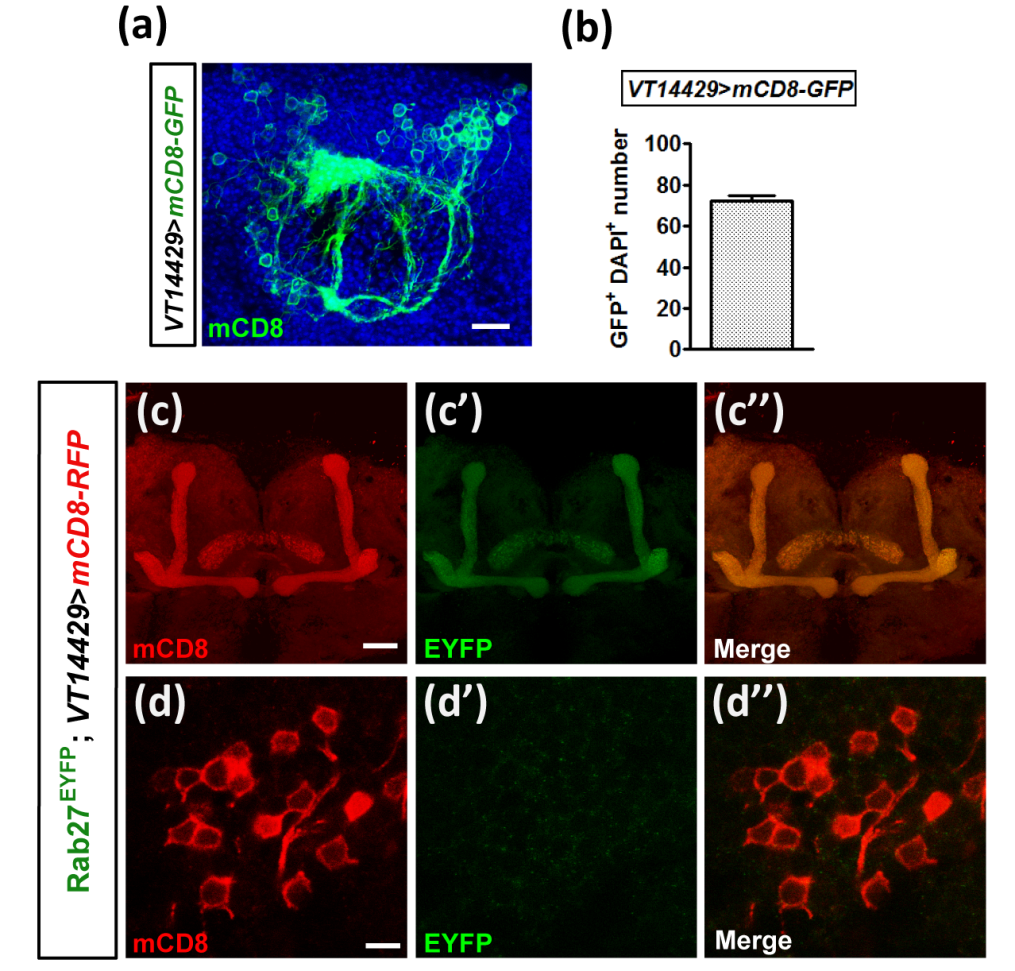
**

**Figure S7**

**
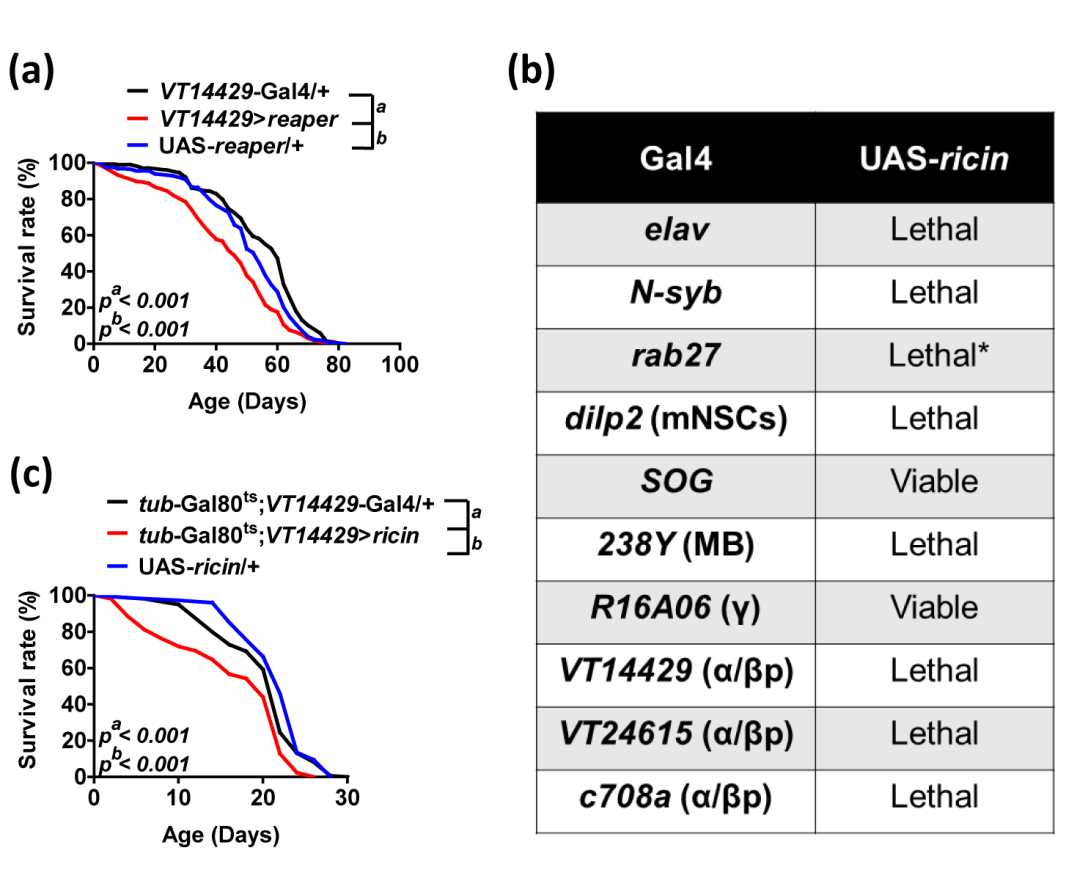
**

**Figure S8**

**
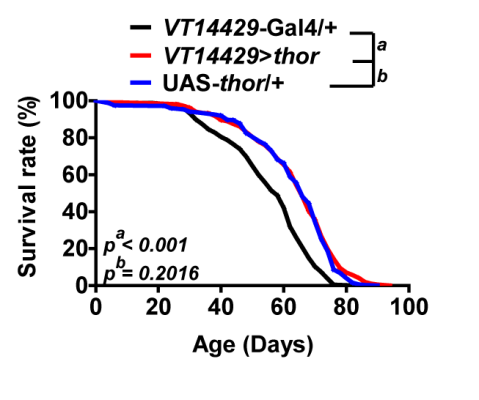
**

**Figure S9**

**
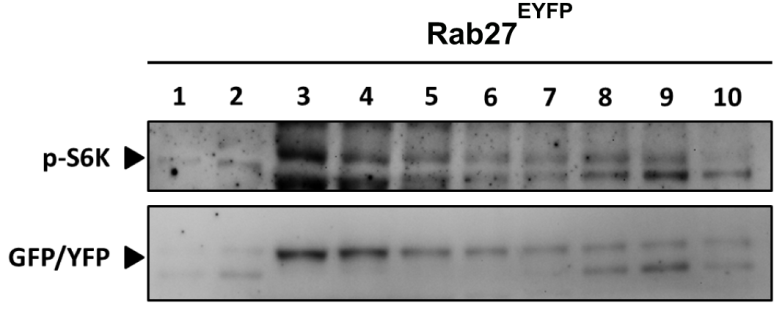
**

**Figure S10**

**
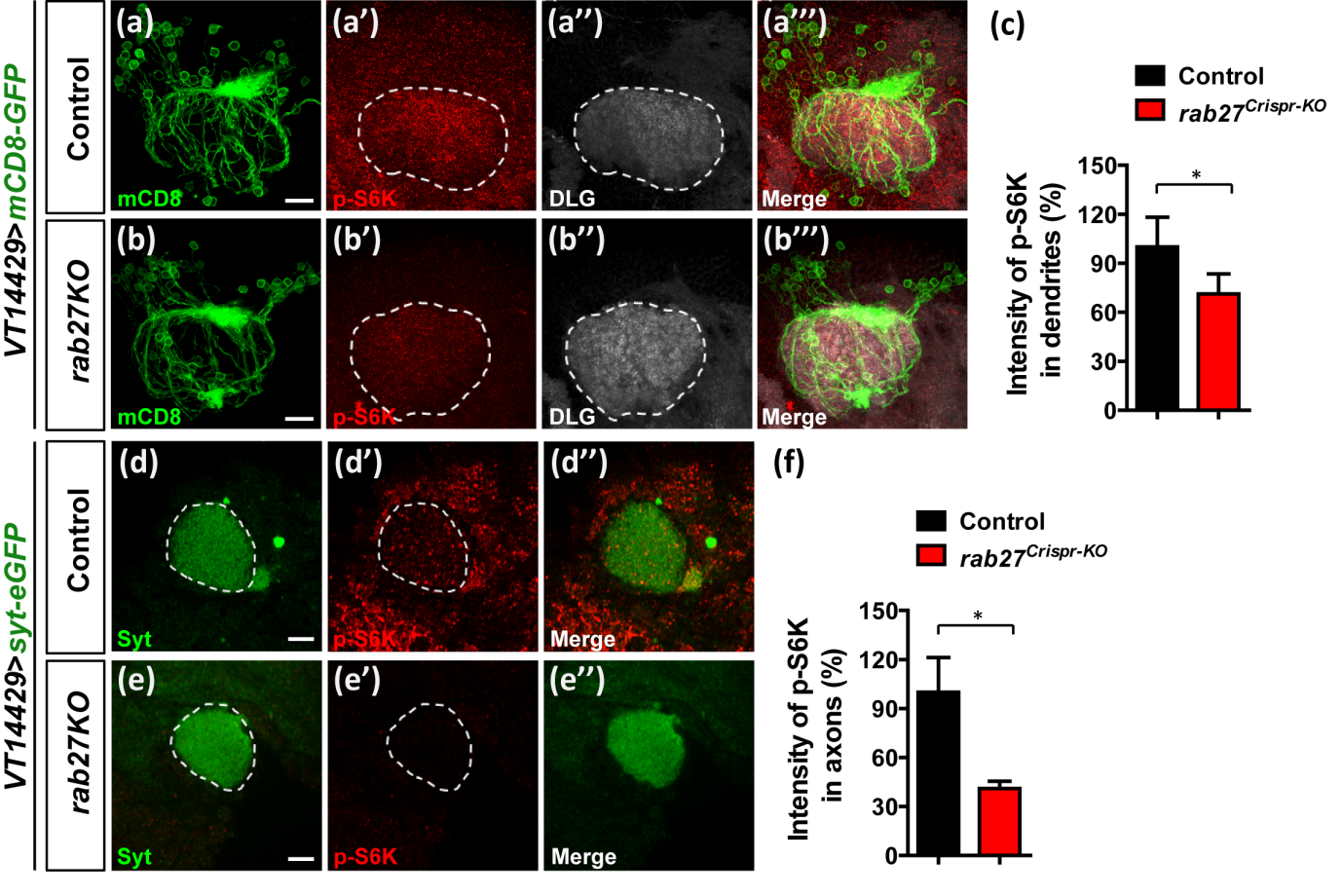
**

**Figure S11**

**
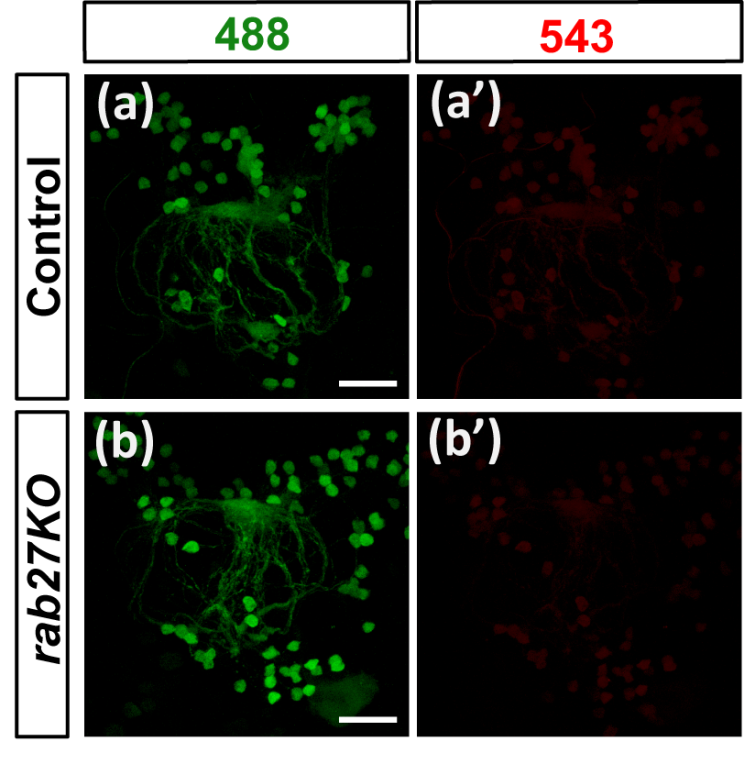
**

**Figure S12**

**
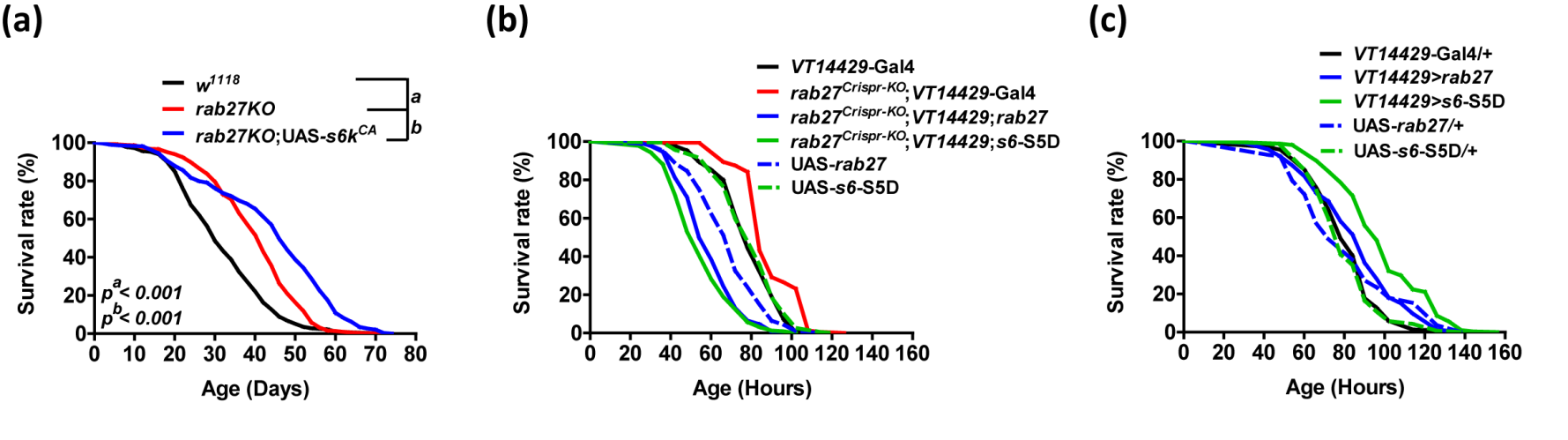
**

**Table S1. The mean lifespan of females for each experimental group compared to the corresponding control(s) in the present study**

|  | Genotype | Mean lifespan (Days) | Compare to: | Change (%) | *p* value | *n* |
| --- | --- | --- | --- | --- | --- | --- |
|  |  |  |  |  |  |  |
| Fig. 1a | *w^1118^* | 30.5 | - | | | 261 |
|  | *rab27^Gal4-KO^*^+/-^ | 36.7 | *w^1118^* | 20.3 | < 0.001 | 260 |
|  | *rab27^Gal4-KO^* | 44.7 | *w^1118^* | 46.6 | < 0.001 | 266 |
|  |  |  | *rab27^Gal4-KO^*^+/-^ | 21.8 | < 0.001 |  |
| Fig. 1c | *w^1118^* | 30.3 | - | | | 2298 |
|  | *rab27^Crispr-KO^* | 46.5 | *w^1118^* | 53.5 | < 0.001 | 432 |
|  | *rab27^Gal4-KO^* | 45.0 | *w^1118^* | 48.5 | < 0.001 | 2140 |
|  | *rab27^Gal4-KO^*;UAS-*rab27* | 34.6 | *rab27^Gal4-KO^* | -23.1 | < 0.001 | 474 |
|  | UAS-*rab27* | 28.1 | - | | | 174 |
| Fig. 1h | EtOH | 37.5 | - | | | 245 |
|  | RU486 | 43.9 | 17.1 | | < 0.001 | 241 |
| Fig. S1c | *w^1118^* | 42.8 | - | | | 98 |
|  | *rab27^Crispr-KO^* | 61 | *w^1118^* | 42.5 | < 0.001 | 96 |
|  | *rab27^Gal4-KO^* | 67.4 | *w^1118^* | 57.5 | < 0.001 | 113 |
| Fig. S2f | *elav*-GS>*rab27*-RNAi (EtOH) | 180.6  hours | - | | | 186 |
|  | *elav*-GS>*rab27*-RNAi (RU486) | 198.1  hours | *elav*-GS>*rab27*-RNAi (EtOH) | 9.7 | < 0.001 | 189 |
|  | *elav*-GS-Gal4/+  (EtOH) | 143.2  hours | - | | | 161 |
|  | *elav*-GS-Gal4/+ ( RU486) | 148.3  hours | *elav*-GS-Gal4/+  (EtOH) | 3.6 | = 0.0114 | 151 |
|  | UAS-*rab27*-RNAi/+ (EtOH) | 138  hours | - | | | 115 |
|  | UAS-*rab27*-RNAi/+ (RU486) | 138.1  hours | UAS-*rab27*-RNAi/+ (EtOH) | 0.1 | = 0.9201 | 121 |
| Fig. 2a | *w^1118^* | 39.6 hours | - | | | 119 |
|  | *rab27^Gal4-KO^* | 47.7 hours | 20.5 | | < 0.001 | 140 |
| Fig. 2b | *w^1118^* | 15.3 hours | - | | | 99 |
|  | *rab27^Gal4-KO^* | 35.7 hours | 133.3 | | < 0.001 | 96 |
| Fig. S3a | Canton-S | 43.4  hours | - | | | 351 |
|  | *rab27^Gal4-KO^* | 52.3  hours | 20.5 | | < 0.001 | 350 |
| Fig. S3c | *w^1118^*  (tetracycline) | 41.0  hours | - | | | 249 |
|  | *rab27^Gal4-KO^* (tetracycline) | 54.0  hours | 31.7 | | < 0.001 | 253 |
| Fig. 3a’ | *238Y-*Gal4/*+* | 28.8 | - | | | 194 |
|  | *238Y>rab27-*RNAi | 35.5 | *238Y-*Gal4/*+* | 23.3 | < 0.001 | 175 |
|  |  |  | UAS*-rab27-*RNAi */+* | 36.5 | < 0.001 |  |
|  | UAS*-rab27-*RNAi*/+* | 26.0 | - | | | 328 |
| Fig. 3b’ | *dilp2-*Gal4*/+* | 32.8 | - | | | 296 |
|  | *dilp2>rab27-*RNAi | 31.1 | *dilp2-*Gal4*/+* | -5.2 | < 0.001 | 450 |
|  |  |  | UAS*-rab27-*RNAi */+* | -6.6 | < 0.01 |  |
|  | UAS*-rab27-*RNAi*/+* | 33.3 | - | | | 387 |
| Fig. 3c’ | *VT49246-*Gal4*/+* | 41.8 | - | | | 217 |
|  | *VT49246>rab27-*RNAi | 49.1 | *VT49246-*Gal4*/+* | 17.5 | < 0.001 | 210 |
|  |  |  | UAS*-rab27-*RNAi */+* | 33.1 | < 0.001 |  |
|  | UAS*-rab27-*RNAi*/+* | 36.9 | - | | | 160 |
| Fig. 3d’ | *VT14429-*Gal4*/+* | 29.5 | - | | | 677 |
|  | *VT14429>rab27-*RNAi | 38.3 | *VT14429-*Gal4*/+* | 29.8 | < 0.001 | 732 |
|  |  |  | UAS-*rab27-*RNAi */+* | 13.0 | *<* 0.001 |  |
|  | UAS*-rab27-*RNAi*/+* | 33.9 | - | | | 187 |
| Fig.  3f | *VT14429>mCD8*-*GFP* | 48.9 | - | | | 352 |
|  | *VT14429>rab27* | 57.2 | *rab27^Crispr-KO^;VT14429>rab27* | 12.4 | < 0.001 | 206 |
|  | *rab27^Crispr-KO^;VT14429>mCD8*-*GFP* | 62.4 | *VT14429>mCD8*-*GFP* | 27.6 | < 0.001 | 688 |
|  | *rab27^Crispr-KO^;VT14429>rab27* | 50.9 | *rab27^Crispr-KO^;VT14429>mCD8*-*GFP* | -18.4 | < 0.001 | 781 |
|  | UAS*-rab27/+* | 55.1 | - | | | 214 |
| Fig.  S5a’ | *rab27-*Gal4*/+* | 36.2 | - | | | 518 |
|  | *rab27>rab27-*RNAi | 40.8 | *rab27-*Gal4*/+* | 12.7 | < 0.001 | 309 |
|  |  |  | UAS*-rab27-*RNAi */+* | 30.4 | < 0.001 |  |
|  | UAS*-rab27-*RNAi*/+* | 31.3 | - | | | 454 |
| Fig.  S5b’ | *SOG-*Gal4*/+* | 46 | - | | | 440 |
|  | *SOG>rab27-*RNAi | 41.8 | *SOG-*Gal4*/+* | -9.1 | < 0.001 | 235 |
|  |  |  | UAS*-rab27-*RNAi */+* | -14.5 | < 0.001 |  |
|  | UAS*-rab27-*RNAi*/+* | 48.9 | - | | | 298 |
| Fig.  S5c’ | *G0050-*Gal4*/+* | 40.6 | - | | | 394 |
|  | *G0050>rab27-*RNAi | 30.9 | *G0050-*Gal4*/+* | -23.9 | < 0.001 | 200 |
|  |  |  | UAS*-rab27-*RNAi */+* | 2.0 | *=* 0.8894 |  |
|  | UAS*-rab27-*RNAi*/+* | 30.3 | - | | | 307 |
| Fig.  S5d’ | *R16A06-*Gal4*/+* | 56.4 | - | | | 230 |
|  | *R16A06>rab27-*RNAi | 42.7 | *R16A06-*Gal4*/+* | -24.3 | < 0.001 | 312 |
|  |  |  | UAS*-rab27-*RNAi */+* | -2.1 | *<* 0.01 |  |
|  | UAS*-rab27-*RNAi*/+* | 43.6 | - | | | 251 |
| Fig. 4a | *VT14429-*Gal4*/+* | 51 | - | | | 912 |
|  | *VT14429>NaCh* | 45.7 | *VT14429-*Gal4*/+* | -10.4 | < 0.05 | 696 |
|  |  |  | UAS*-NaCh/+* | -20.7 | < 0.001 |  |
|  | UAS*-NaCh/+* | 57.6 | - | | | 377 |
| Fig.  S7a | *VT14429-*Gal4*/+* | 54.6 | - | | | 274 |
|  | *VT14429>reaper* | 43.1 | *VT14429-*Gal4*/+* | -21.1 | < 0.001 | 263 |
|  |  |  | UAS*-reaper/+* | -14.7 | < 0.001 |  |
|  | UAS*-reaper**/+* | 50.5 | - | | | 248 |
| Fig.  S7c | *tub*-Gal80^ts^;*VT14429-*Gal4 | 20.1 | - | | | 159 |
|  | *tub*-Gal80^ts^;*VT14429*>*ricin* | 16.3 | *tub*-Gal80^ts^;*VT14429-*Gal4 | -18.9 | < 0.001 | 165 |
|  |  |  | UAS-*ricin/+* | -24.5 | < 0.001 |  |
|  | UAS-*ricin/+* | 21.6 | - | | | 74 |
| Fig. 5a | *w^1118^* (EtOH) | 21.3 |  | | | 210 |
|  | *w^1118^* (Rapamycin) | 28.4 | *w^1118^* (EtOH) | 33.3 | < 0.001 | 206 |
|  | *rab27KO* (EtOH) | 41 | *w^1118^* (EtOH) | 92.5 | < 0.001 | 331 |
|  |  |  | *rab27KO* (Rapamycin) | -0.2 | *=* 0.3423 |  |
|  | *rab27KO* (Rapamycin) | 41.1 | - | | | 242 |
| Fig. 5b | *w^1118^* | 33.1 | - | | | 666 |
|  | *rab27^Gal4-KO^* | 45.2 | *w^1118^* | 36.6 | < 0.001 | 450 |
|  | *rab27^Gal4-KO^*;UAS-*tsc2* | 46.1 | *rab27^Gal4-KO^* | 2.0 | *=* 0.7822 | 714 |
|  | UAS-*tsc2* | 35.2 | - | | | 157 |
| Fig. 5c | *rab27-*Gal4*/+* | 44.2 | - | | | 518 |
|  | *rab27>s6k^DN^* | 56.2 | *rab27-*Gal4*/+* | 27.1 | < 0.001 | 609 |
|  |  |  | UAS-*s6k^DN^/+* | 5.2 | < 0.001 |  |
|  | UAS-*s6k^DN^/+* | 53.4 | - | | | 288 |
| Fig.  5d | *VT14429-*Gal4*/+* | 46.6 | - | | | 1140 |
|  | *VT14429>s6k^DN^* | 61.9 | *VT14429-*Gal4*/+* | 32.8 | < 0.001 | 774 |
|  |  |  | UAS-*s6k^DN^/+* | 14.2 | < 0.001 |  |
|  | UAS-*s6k^DN^/+* | 54.2 | - | | | 355 |
| Fig.  S8 | *VT14429-*Gal4*/+* | 54.7 | - | | | 394 |
|  | *VT14429>thor* | 63.2 | *VT14429-*Gal4*/+* | 15.5 | < 0.001 | 367 |
|  |  |  | UAS-*thor/+* | 1.1 | = 0.2016 |  |
|  | UAS-*thor/+* | 62.5 | - | | | 322 |
| Fig.  S12a | *w^1118^* | 31.9 | - | | | 525 |
|  | *rab27^Gal4-KO^* | 39.9 | *w^1118^* | 25.1 | < 0.001 | 496 |
|  |  |  | *rab27^Gal4-KO^*;UAS-*s6k^CA^* | -9.3 | < 0.001 |  |
|  | *rab27^Gal4-KO^*;UAS-*s6k^CA^* | 44.0 | - | | | 568 |
| Fig.  S12b | *VT14429-*Gal4 | 78.7  hours | - | | | 169 |
|  | *rab27^Crispr-KO^;VT14429* | 89.0  hours | *VT14429-*Gal4 | 13.1 | < 0.001 | 151 |
|  | *rab27^Crispr-KO^;VT14429>rab27* | 58.9  hours | *rab27^Crispr-KO^;VT14429* | -33.8 | < 0.001 | 261 |
|  | *rab27^Crispr-KO^;VT14429>s6*-S5D | 55.0  hours | *rab27^Crispr-KO^;VT14429* | -38.2 | < 0.001 | 227 |
|  | UAS-*rab27* | 68.3  hours | - | | | 188 |
|  | UAS-*s6*-S5D | 79.0  hours | - | | | 188 |
| Fig.  S12c | *VT14429-*Gal4*/+* | 80.5  hours | - | | | 220 |
|  | *VT14429>rab27* | 85.3  hours | *VT14429-*Gal4*/+* | 6.0 | < 0.001 | 298 |
|  |  |  | UAS-*rab27*/+ | 6.0 | = 0.4379 |  |
|  | *VT14429>s6*-S5D | 98.2  hours | *VT14429-*Gal4*/+* | 22.0 | < 0.001 | 290 |
|  |  |  | UAS-*s6*-S5D/+ | 23.1 | < 0.001 |  |
|  | UAS-*rab27*/+ | 80.5  hours | - | | | 111 |
|  | UAS-*s6*-S5D/+ | 79.8  hours | - | | | 140 |

**Table S2. The mean lifespan of males for each experimental group compared to the corresponding control(s) in the present study**

|  | Genotype | Mean lifespan (Days) | Compare to: | Change (%) | *p* value | *n* |
| --- | --- | --- | --- | --- | --- | --- |
|  |  |  |  |  |  |  |
| Fig. 1c | *w^1118^* | 35.5 | - | | | 2318 |
|  | *rab27^Crispr-KO^* | 52.6 | *w^1118^* | 48.2 | < 0.001 | 876 |
|  | *rab27^Gal4-KO^* | 52.8 | *w^1118^* | 48.7 | < 0.001 | 2209 |
|  | *rab27^Gal4-KO^*;UAS-*rab27* | 32.4 | *rab27^Gal4-KO^* | -38.6 | < 0.001 | 417 |
|  | UAS-*rab27* | 32.2 | - | | | 237 |
| Fig. 1h | EtOH | 43.0 | - | | | 399 |
|  | RU486 | 44.8 | 4.2 | | < 0.01 | 356 |
| Fig. 3a’ | *238Y-*Gal4/*+* | 29.4 | - | | | 131 |
|  | *238Y>rab27-*RNAi | 37.8 | *238Y-*Gal4/*+* | 28.6 | < 0.001 | 216 |
|  |  |  | UAS*-rab27-*RNAi */+* | 21.5 | < 0.001 |  |
|  | UAS*-rab27-*RNAi*/+* | 31.1 | - | | | 281 |
| Fig. 3b’ | *dilp2-*Gal4*/+* | 47.6 | - | | | 348 |
|  | *dilp2>rab27-*RNAi | 38.6 | *dilp2-*Gal4*/+* | -18.9 | < 0.001 | 419 |
|  |  |  | UAS*-rab27-*RNAi */+* | -2.5 | *=* 0.3141 |  |
|  | UAS*-rab27-*RNAi*/+* | 39.6 | - | | | 424 |
| Fig. 3c’ | *VT49246-*Gal4*/+* | 40.4 | - | | | 273 |
|  | *VT49246>rab27-*RNAi | 50.6 | *VT49246-*Gal4*/+* | 25.2 | < 0.001 | 232 |
|  |  |  | UAS*-rab27-*RNAi */+* | 39.0 | < 0.001 |  |
|  | UAS*-rab27-*RNAi*/+* | 36.4 | - | | | 181 |
| Fig. 3d’ | *VT14429-*Gal4*/+* | 38.3 | - | | | 669 |
|  | *VT14429>rab27-*RNAi | 42.5 | *VT14429-*Gal4*/+* | 11.0 | < 0.001 | 773 |
|  |  |  | UAS-*rab27-*RNAi */+* | 21.1 | *<* 0.001 |  |
|  | UAS*-rab27-*RNAi*/+* | 35.1 | - | | | 236 |
| Fig.  S5a’ | *rab27-*Gal4*/+* | 40.8 | - | | | 433 |
|  | *rab27>rab27-*RNAi | 44.4 | *rab27-*Gal4*/+* | 8.8 | < 0.001 | 317 |
|  |  |  | UAS*-rab27-*RNAi */+* | 42.8 | < 0.001 |  |
|  | UAS*-rab27-*RNAi*/+* | 31.1 | - | | | 281 |
| Fig.  S5b’ | *SOG-*Gal4*/+* | 50.9 | - | | | 352 |
|  | *SOG>rab27-*RNAi | 43.9 | *SOG-*Gal4*/+* | -13.8 | < 0.001 | 226 |
|  |  |  | UAS*-rab27-*RNAi */+* | -13.1 | < 0.001 |  |
|  | UAS*-rab27-*RNAi*/+* | 50.5 | - | | | 307 |

| Fig.  S5c’ | *G0050-*Gal4*/+* | 35.0 | - | | | 113 |
| --- | --- | --- | --- | --- | --- | --- |
|  | *G0050>rab27-*RNAi | 28.2 | *G0050-*Gal4*/+* | -19.4 | < 0.001 | 121 |
|  |  |  | UAS*-rab27-*RNAi */+* | -16.6 | < 0.001 |  |
|  | UAS*-rab27-*RNAi*/+* | 33.8 | - | | | 300 |
| Fig.  S5d’ | *R16A06-*Gal4*/+* | 53.6 | - | | | 196 |
|  | *R16A06>rab27-*RNAi | 44.6 | *R16A06-*Gal4*/+* | -16.8 | < 0.001 | 305 |
|  |  |  | UAS*-rab27-*RNAi */+* | 11.8 | *<* 0.01 |  |
|  | UAS*-rab27-*RNAi*/+* | 39.9 | - | | | 300 |
| Fig. 4a | *VT14429-*Gal4*/+* | 54.5 | - | | | 888 |
|  | *VT14429>NaCh* | 52.5 | *VT14429-*Gal4*/+* | -3.7 | < 0.001 | 674 |
|  |  |  | UAS*-NaCh/+* | -17.3 | < 0.001 |  |
|  | UAS*-NaCh/+* | 63.5 | - | | | 530 |
| Fig.  S7a | *VT14429-*Gal4*/+* | 54.0 | - | | | 291 |
|  | *VT14429>reaper* | 34.0 | *VT14429-*Gal4*/+* | -37.0 | < 0.001 | 320 |
|  |  |  | UAS*-reaper/+* | -40.3 | < 0.001 |  |
|  | UAS*-reaper/+* | 57.0 | - | | | 302 |
| Fig.  S7c | *tub*-Gal80^ts^;*VT14429-*Gal4 | 20.4 | - | | | 93 |
|  | *tub*-Gal80^ts^;*VT14429*>*ricin* | 17.3 | *tub*-Gal80^ts^;*VT14429-*Gal4 | -15.2 | < 0.001 | 67 |
|  |  |  | UAS-*ricin/+* | -25.4 | < 0.001 |  |
|  | UAS-*ricin/+* | 23.2 | - | | | 74 |
| Fig. 5a | *w^1118^* (EtOH) | 36.9 |  | | | 196 |
|  | *w^1118^* (Rapamycin) | 39.3 | *w^1118^* (EtOH) | 6.5 | < 0.001 | 157 |
|  | *rab27KO* (EtOH) | 44.9 | *w^1118^* (EtOH) | 21.7 | < 0.001 | 225 |
|  |  |  | *rab27KO* (Rapamycin) | 6.7 | *=* 0.2229 |  |
|  | *rab27KO* (Rapamycin) | 42.1 | - | | | 138 |
| Fig. 5b | *w^1118^* | 38.6 | - | | | 605 |
|  | *rab27^Gal4-KO^* | 49.9 | *w^1118^* | 29.3 | < 0.001 | 541 |
|  | *rab27^Gal4-KO^*;UAS-*tsc2* | 48.4 | *rab27^Gal4-KO^* | 3.0 | < 0.01 | 545 |
|  | UAS-*tsc2* | 39.0 | - | | | 230 |
| Fig. 5c | *rab27-*Gal4*/+* | 47.6 | - | | | 466 |
|  | *rab27>s6k^DN^* | 56.0 | *rab27-*Gal4*/+* | 17.6 | < 0.001 | 583 |
|  |  |  | UAS-*s6k^DN^/+* | 13.8 | < 0.001 |  |
|  | UAS-*s6k^DN^/+* | 49.2 | - | | | 163 |
| Fig.  5d | *VT14429-*Gal4*/+* | 49.0 | - | | | 792 |
|  | *VT14429>s6k^DN^* | 59.9 | *VT14429-*Gal4*/+* | 22.2 | < 0.001 | 631 |
|  |  |  | UAS-*s6k^DN^/+* | 7.9 | < 0.001 |  |
|  | UAS-*s6k^DN^/+* | 55.5 | - | | | 289 |
| Fig.  S8 | *VT14429-*Gal4*/+* | 53.9 | - | | | 428 |
|  | *VT14429>thor* | 61.3 | *VT14429-*Gal4*/+* | 13.7 | < 0.001 | 429 |
|  |  |  | UAS-*thor/+* | 0.2 | = 0.4696 |  |
|  | UAS-*thor/+* | 61.2 | - | | | 437 |
| Fig.  S12a | *w^1118^* | 40.3 | - | | | 503 |
|  | *rab27^Gal4-KO^* | 51.4 | *w^1118^* | 27.5 | < 0.001 | 510 |
|  |  |  | *rab27^Gal4-KO^*;UAS-*s6k^CA^* | 3.4 | = 0.1567 |  |
|  | *rab27^Gal4-KO^*;UAS-*s6k^CA^* | 49.7 | - | | | 643 |

Lifespan differences are listed as the percentages of extension compared with the indicated control. The *p* values were obtained using the log-rank test. *n* number indicates the number of flies tested.

**REFERENCE**

Grobler, Y., Yun, C. Y., Kahler, D. J., Bergman, C. M., Lee, H., Oliver, B., & Lehmann, R. (2018). Whole genome screen reveals a novel relationship between Wolbachia levels and Drosophila host translation. *PLOS Pathogens, 14*(11), e1007445. doi: 10.1371/journal.ppat.1007445
